# An Estimation of Erinaceidae Phylogeny: A Combined Analysis Approach

**DOI:** 10.1371/journal.pone.0039304

**Published:** 2012-06-20

**Authors:** Kai He, Jian-Hai Chen, Gina C. Gould, Nobuyuki Yamaguchi, Huai-Sen Ai, Ying-Xiang Wang, Ya-Ping Zhang, Xue-Long Jiang

**Affiliations:** 1 State Key Laboratory of Genetic Resources and Evolution, Kunming Institute of Zoology, Chinese Academy of Sciences, Kunming, Yunnan, China; 2 Graduate University of Chinese Academy of Sciences, Beijing, China; 3 University of Connecticut, Department of Ecology and Evolutionary Biology, Stamford, Connecticut, United States of America; 4 Bruce Museum, Greenwich, Connecticut, United States of America; 5 Department of Biological & Environmental Sciences, Qatar University, Doha, Qatar; 6 Baoshan Administrative Bureau, Gaoligong National Nature Reserve, Baoshan, China; Barnard College, Columbia University, United States of America

## Abstract

**Background:**

Erinaceidae is a family of small mammals that include the spiny hedgehogs (Erinaceinae) and the silky-furred moonrats and gymnures (Galericinae). These animals are widely distributed across Eurasia and Africa, from the tundra to the tropics and the deserts to damp forests. The importance of these animals lies in the fact that they are the oldest known living placental mammals, which are well represented in the fossil record, a rarity fact given their size and vulnerability to destruction during fossilization. Although the Family has been well studied, their phylogenetic relationships remain controversial. To test previous phylogenetic hypotheses, we combined molecular and morphological data sets, including representatives of all the genera.

**Methodology and Principal Findings:**

We included in the analyses 3,218 bp mitochondrial genes, one hundred and thirty-five morphological characters, twenty-two extant erinaceid taxa, and five outgroup taxa. Phylogenetic relationships were reconstructed using both partitioned and combined data sets. As in previous analyses, our results strongly support the monophyly of both subfamilies (Galericinae and Erinaceinae), the *Hylomys* group (to include *Neotetracus* and *Neohylomys*), and a sister-relationship of *Atelerix* and *Erinaceus*. As well, we verified that the extremely long branch lengths within the Galericinae are consistent with their fossil records. Not surprisingly, we found significant incongruence between the phylogenetic signals of the genes and the morphological characters, specifically in the case of *Hylomys parvus, Mesechinus*, and relationships between *Hemiechinus* and *Paraechinus*.

**Conclusions:**

Although we discovered new clues to understanding the evolutionary relationships within the Erinaceidae, our results nonetheless, strongly suggest that more robust analyses employing more complete taxon sampling (to include fossils) and multiple unlinked genes would greatly enhance our understanding of the Erinaceidae. Until then, we have left the nomenclature of the taxa unchanged; hence it does not yet precisely reflect their phylogenetic relationships or the depth of their genetic diversity.

## Introduction

**Figure 1:**
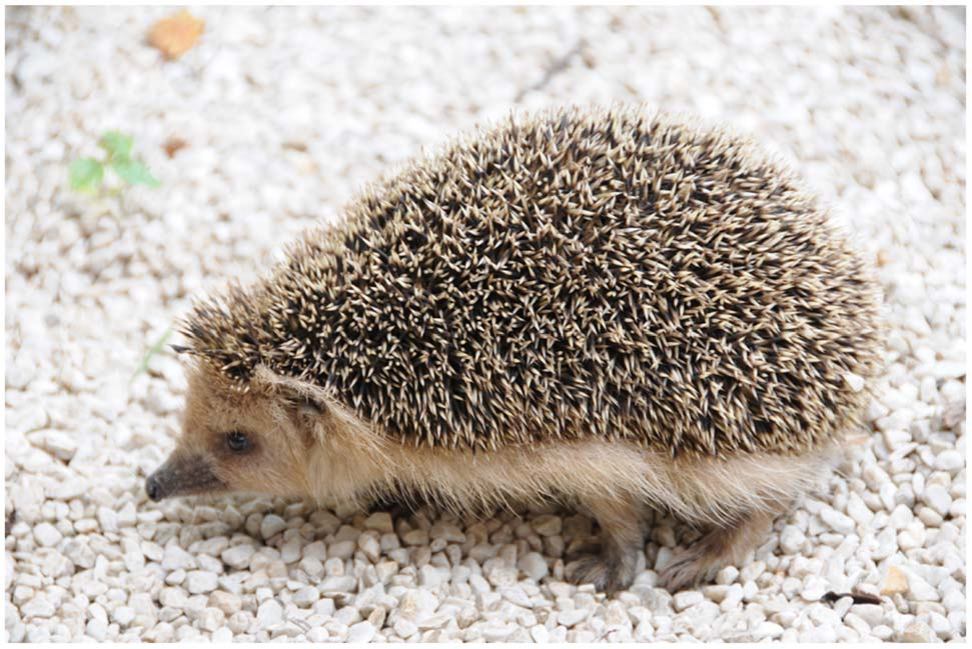
A Living hedgehog. Photograph of the Daurian Hedgehog (*Mesechinus dauuricus*) from Liaoning, China.

**Figure 2:**
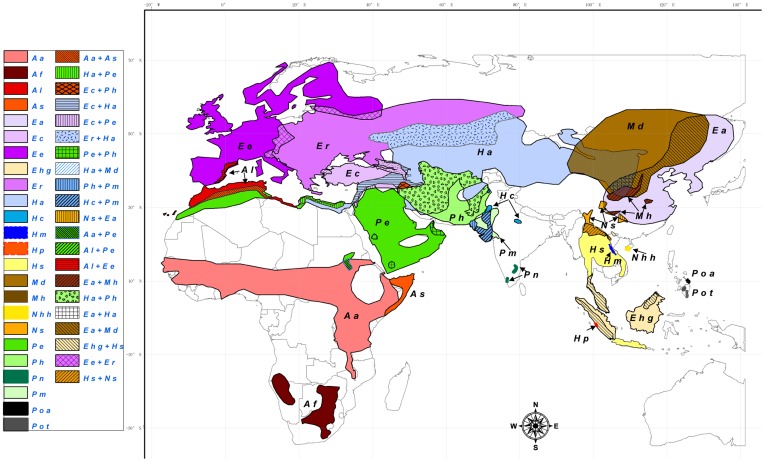
Distribution of the Family Erinaceidae and its component species. Modified based on Corbet et al. [1] and IUCN red list (iucnredlist.org). Galericinae: Ehg, *Echinosorex gymnura*; Hm, *Hylomys megalotis*; Hp, *Hylomys parvus*; Hs, *Hylomys suillus*; Nhh, *Neohylomys hainanensis*; NS, *Neotetracus sinensis*; Poa, *Podogymnura aureospinula*; Pot, *Podogymnura truei*. Erinaceinae: Aa, *Atelerix albiventris*; Af, *Atelerix frontalis*; Al, *Atelerix algirus*; As, *Atelerix sclateri*; Ea, *Erinaceus amurensis*; Ec, *Erinaceus concolor*; Ee, *Erinaceus europaeus*; Er, *Erinaceus roumanicus*; Ha, *Hemiechinus auritus*; Hc, *Hemiechinus collaris*; Md, *Mesechinus dauuricus*; Mh, *Mesechinus hughi*; Pe, *Paraechinus aethiopicus*; Ph, *Paraechinus hypomelas*; Pn, *Paraechinus nudiventris*; Pm, *Paraechinus micropus*.

**Figure 3:**
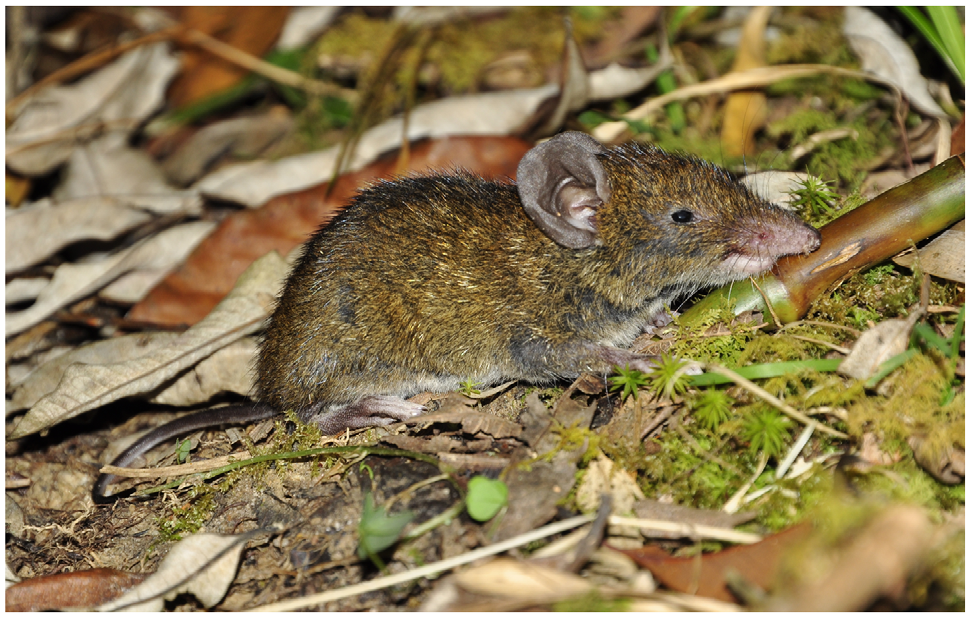
A living gymnure. Photograph of the Shrew Gymnure (*Neotetracus sinensis*) from Yunnan, China.

Sometimes confused with porcupines, hedgehogs (erinaceines) are small, spiny nocturnal mammals (Figure 1) that live throughout Eurasia and Africa (Figure 2). Hedgehog habitats extend from the deserts to the tropics [1] and most can hibernate/torpor when the climate gets cold, or in times of food scarcity [2]. The closest living relatives to hedgehogs are the moonrats and gymnures (galericines). Unlike hedgehogs, they are silky-skinned (Figure 3), they are incapable of hibernating [3], and their distribution is confined to the damp forests in Southeast Asia [1] (Figure 2). These two subfamilies are within the Family Erinaceidae, an enigmatic group that has been problematic for evolutionary biologists for decades.

The importance of these small mammals lies within the fact that they are the oldest (known) living placental mammals, which are chronicled by a robust fossil history extending back to the early Paleocene of North America [4]. By then, erinaceomorphs (early relatives to erinaceids) were already recognizable as such, sporting the most definable characteristics of the group–the presence of a prevailed shear of the P4/M1 and expanded talonids and trigonids. Extrapolating into the past, erinaceomorphs most likely split from their sister group sometime in the Late Cretaceous, approximately seventy million years ago [5], [6], [7], [8]. They have diversified and dispersed since then. Fossil erinaceids have been found all over the world except for South America and Australia [9], ranging in size from very small to that of a Jack Russell Terrier [10]. Today, extant hedgehogs and moonrats are limited to Europe, Asia, and Africa; they went extinct in North America some five million years ago [11].

**Figure 4:**
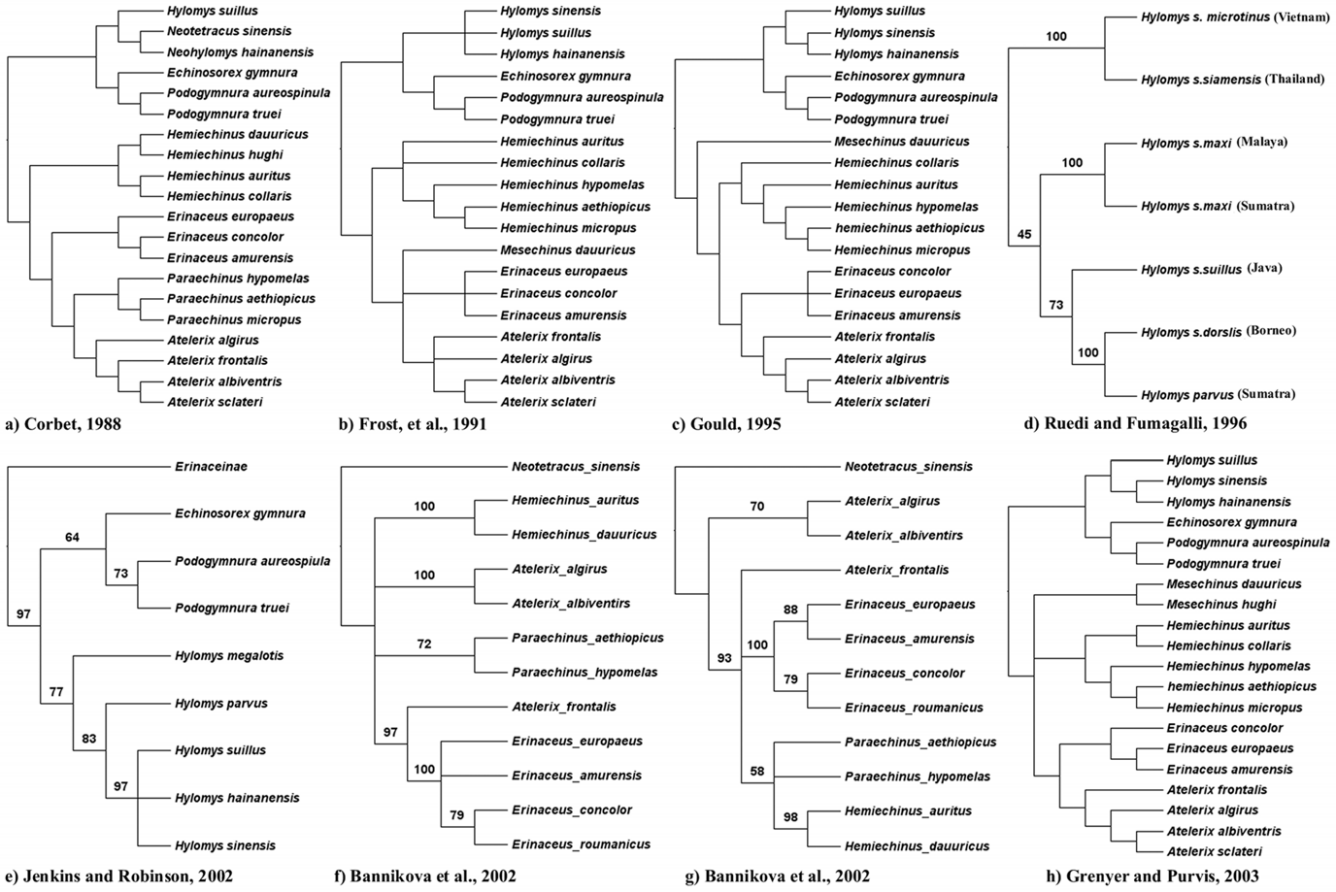
Previous phylogenetic hypotheses. Phylogenetic relationships of Erinaceidae proposed by: (a) Corbet [1]; (b) Frost et al. [17]; (c) Ruedi and Fumagalli [16]; (d) Jenkins and Robinson [13]; (e) and (f) Bannikova et al. [14]; (g) Grenyer and Purvis [12].

Despite the fact that there is a fairly robust, well-studied fossil record for erinaceids, the intra-family phylogenetic relationships are still controversial. A number of alternative phylogenies have been proposed based on morphological characters or short interspersed elements (SINEs) [1], [5], [12], [13], [14], [15], [16], [17] (Figure 4). Discrepancies between phylogenies are predominately relegated to the terminal branches within the family tree, not entire clades (groups of branches), which is further compounded by disparate sampling of taxa and characters, for example: no genetic sequences have been published for the genera *Mesechinus* and *Neotetracus* and only a few short genes are available for *Neohylomys* and *Paraechinus.*

Morphological character sampling is as well disparate. Museum specimens of living taxa are distributed across three continents (North America, Europe, and Asia). This also holds true for the fossil taxa, hence inclusion of the majority of known taxa has been constrained by access. Taxon sampling is further compounded by inconsistent preservation. Not all specimens are complete, in many cases the postcranial material was not preserved together with the skins and skulls, and fossil material is incomplete by its very nature.

To date, DNA sequences have not been considered broadly, nor have molecular and morphological datasets been readily combined to investigate erinaceid phylogeny. Consequently, it is not yet possible to fully understand how each character set recalls the evolutionary history of erinaceids.

In this paper, we combine two robust datasets: mitochondrial DNA for fourteen of the twenty-four living species, and one hundred and thirty-five morphological characters for twenty-two species, including a new species (*Mesechinus hughi*) from China. Three mitochondrial genes including complete 12S rRNA, Cytochrome B (*CYT B*) and NADH dehydrogenase subunit 2 (*ND2*) were obtained, all of which have long been widely employed in mammal systematics and have been demonstrated to have strong phylogenetic signals. Sequences of gymnures (*Hylomys, Neohylomys and Neotetracus*) and hedgehogs (*Erinaceus, Hemiechinus, Mesechinus and Paraechinus*) were obtained. With additional sequences from GenBank, all of the erinaceid genera were sampled making it possible to compare the phylogenetic information of the data sets and then perform a combined analysis.

Although there are many fossil taxa that should/need to be included in a phylogenetic analysis, we leave that for a subsequent analysis [18]. This decision is based on the fact that the inherent missing data that fossils present is known to (sometimes) overwhelm what might otherwise be strong phylogenetic signals within the living taxa [19], [20]. Consequently, we thought it pertinent to first begin with the most complete data set available; that is, in terms of the most coded in a data matrix, not necessarily in terms of taxon sampling.

## Results

### Morphological Data

One hundred and thirty-five morphological transformation series (TS; Text S1) for twenty-two species (Table S1) of extant erinaceines were considered (see Methods). The characters listed herein are TS proposed by Corbet [1], Frost et al. [17], Gould [21], Ruedi and Fumagalli [16], among others (see [21] for a complete list). These TS include everything from cranial, postcranial, and pelage characters, to the finer dental characters often used by paleomammalogists to describe fragmentary fossil erinaceid material (see [15] for a comprehensive list). The latter were included to ascertain their phylogenetic signal among extant taxa, and to establish a robust character database for further study in which fossil taxa can be easily assimilated by anyone.

**Figure 5:**
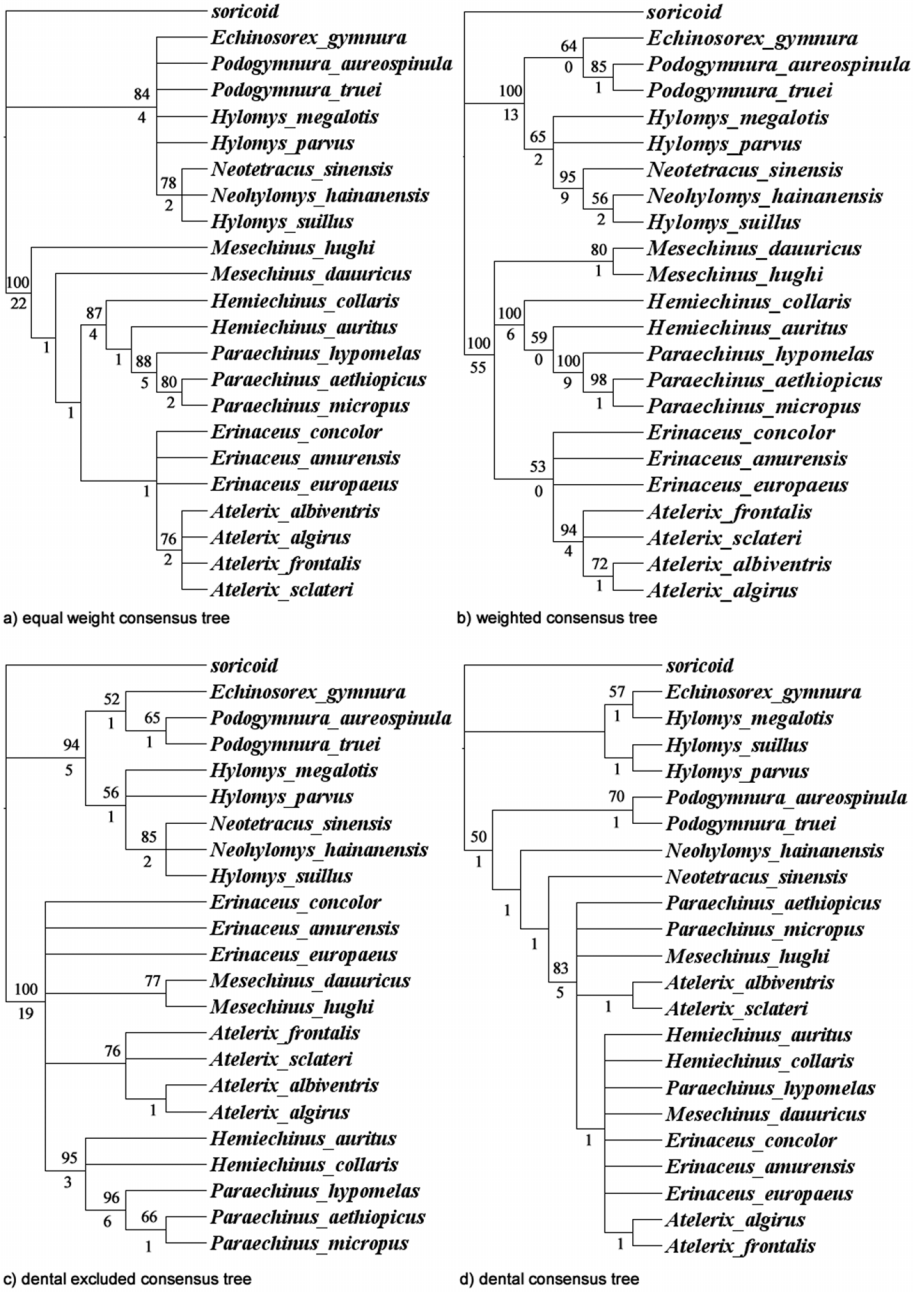
Morphological phylogeny of 22 erinaceids. Morphological strict consensus trees for 23 species using equal weighted (a) and unequal weighted (b), non-dental (c) and dental-only characters (d). Numbers above branches indicate bootstrap values, those below the branches indicate Bremer supports.

Four analyses were conducted on the adjusted one hundred and twelve characters and the twenty-two taxa plus one outgroup. These analyses compared the effects of weighting the cranial characters over the dental characters and partitioning non-dental vs. dental characters. Results of the equally weighted data set recovered 38 trees with a total length of 188 steps, while the weighted data set recovered only four trees. Their strict consensus trees are illustrated in Figure 5a and 5b, respectively. Partitioning the data resulted in the discovery of six trees, 115 steps long when only the non-dental characters were considered; when only the dental characters were considered, 680 trees with a length of 52 steps were recovered, their strict consensus trees are illustrated in Figure 5c and 5d, respectively. These analyses indicate cranial, and the few coded postcranial and pelage characters are critical for recovering both a monophyletic Galericinae and *Mesechinus*. These results are unsurprising because dental characters have been demonstrated to be highly plastic within the Erinaceidae [15] and suggest that weighting characters helps to resolve some clades [22].

The same four analyses were performed on the fifteen taxa, including the outgroup taxon, for which there is genetic data, their strict consensus trees are presented in Figure S1a-d, respectively. These analyses were used to test if taxon sampling accounts for the discrepancies between the gene and morphological trees. The results of weighting and partitioning characters on this subsample of taxa had no effect on the resultant consensus trees.

### Morphological Relationships-Galericinae

The most stymieing issue for this group is the lack of data for the transformation series that define the group: (TS 9) location of the antorbital flange; (TS 121) presence or absence of a post ventral keel on the axis; (TS 122) shape of metacromion process on scapula; (TS 123) shape of the neural spines on the sacral vertebrae; (TS 124) fusion of neural spines of sacral vertebrae; (TS 125) extent of elongation of the posterodorsal process on the ischium; and (TS 126) development of a lateral flange on the tibia (Test S2). Taxa not coded for some of these characters include the podogymnurids, *N. hainanensis*, *H. megalotis*, and *H. parvus*. Because these are the most reliably diagnostic characteristics for the Galericinae, the ability to code for these transformations series would certainly strengthen one hypothesis over another. Missing data values are due to poorly preserved specimens and the fact that postcranial material was not typically preserved with the skulls and skins when the animals were collected.

Even so, based on the morphological characters coded, some conclusions can be put forth for this group: *Hylomys megalotis* presents itself as a species with an interesting combination of derived characteristics that it shares with both the *Echinosorex + Podogymnura* (e.g., TS 4.0; 5.1; 83.0) and the *Hylomys* group (to include *Neotetracus* and *Neohylomys*) (e.g., TS 15.1; 19.1) resulting in *H. megalotis* bouncing around within the galericine clade. Its uniqueness is compounded by the missing data values across several of the most diagnostic transformation series within the Galericinae (see above list). Equally problematic is the considerable missing values for *H. parvus*, many of which overlap with *H. megalotis* (note that these two taxa were coded from the literature, see Morphology section above).

There has been much debate regarding the veracity of the status of *Neotetracus sinensis* and *Neohylomys hainanensis* as separate genera [1], and whether or not they form a monophyletic group with *Hylomys* [5], [12], [13], [17], more specifically, with *H. suillus*. This is because it has been only recently that *H. parvus* and/or *H. megalotis* have been included in a phylogenetic analysis of any kind, morphological [16] or molecular [13]. In this analysis, inclusion of the two species made the situation more complex. Like others [13], [17], *N. sinensis*, *N. hainanensis* and *H. suillus* bounce around within the *Hylomys* clade without any additional steps to the tree. The characters that firmly unite these three taxa in one clade are TS 14.1 (the presence of a distinct supraorbital process, a frontal process on the parietal frontal suture); and TS 16.1 (presence of an anterior process of the parietal which extends anteriorly along the supraorbital rim to form the base of the supraorbital process). None of these characters are either polymorphic or exhibit any kind of reversals within any of the other taxa among the erinaceids. In contrast, the *H. megalotis* and *H. parvus* were found to be basal within the *Hylomys* group in the weighted and non-dental character analyses, rendering *Hylomys* a paraphyletic group. The characters that *H. suillus* shares in common with *H. parvus* and *H. megalotis* are the presence of P_1_ and the size of the P_3._ The presence and absence of P_1_ does not seem to co-vary greatly within erinaceids [15] (Note Gould only looked at twenty-five specimens per species, not a robust sample size by any standard). Even so, this character state is polymorphic in *Echinosorex* and *Paraechinus aethiopicus*.

The expression of the P_3_ is another story. In *H. suillus*, P_3_ exhibits polymorphism [15]: sometimes it has two roots, as exhibited by *H. parvus* and *H. megalotis*, and sometimes it only has one root, as seen in the other two species of the *Hylomys* group. This latter character state, as evidence of the monophyly of *H. suillus*+*H. parvus*+*H. megalotis,* we reject summarily because there is no indication of a robust phylogenetic signal.

The characters that unite *H. parvus+H. megalotis* as sister taxa are TS 21.0, position of the suboptic foramen anterior to the sphenorbital fissure, which they share in common with *Echinosorex* (this transformation can be optimized differently along the tree without additional steps rendering it ambiguous); and the TS 25.1, absence of the posterior palatal shelf, which varies significantly among erinaceids and is suspect.

Because we had to rely on the literature, several important characters could not be coded for *H. parvus* and *H. megalotis*: TS 9, the location of the antorbital flange, which presumably indicates the relative length and motility of the fleshy proboscis of erinaceids; and TS 22, the presence and position of the alisphenoid canal, which portends the overall length of the skull (in erinaceines, as the orbital temporal region shortens, the alisphenoid canal disappears with a shortening of the snout). More data on these two transformation series might make a difference in the overall topology of the tree.

In summary, the morphological data for the *Hylomys* group (to include *Neotetracus* and *Neohylomys*) suggests that the group is monophyletic, but relationships among the species remain obscure.

### Morphological Relationships - Erinaceinae

There has been some debate on whether or not *Hemiechinus* and *Paraechinus* constitute a monophyletic group (e.g., [1] vs. [17]), and if so, are they substantially different from one another to warrant generic distinction? The most recent morphological studies suggest that not only are they a monophyletic group, but that indeed, they should be all subsumed into the genus *Hemiechinus* [5], [12], [17], while molecular studies suggest otherwise [14] (see discussion below under Molecular Data).

In this analysis, like the other more recent morphological analyses, *Paraechinus* and *Hemiechinus* are discovered to be monophyletic (Figure 5a and 5b) and are supported by a suite of cranial (TS 3.1); and highly unusual auditory characters (TS 12.2, 12.3; 30.2, 30.3, 30.4; and TS 34.1) (Test S2). This series of characteristics are related to the progressive inflation of the ear region: inflation of the pterygoid/alisphenoid and eptiterygoid bones, as well as the mastoid region of the skull, and a deepening of the nasophyrangeal fossa. Definitive, or historical hemechinies (*H. auritus* and *H. collaris)* exhibit the most extreme inflation of the ear region, while *P. aethiopicus*, and *P. micropus*, exhibit less, albeit progressive inflation, respectively. That is, inflation of the entire auditory region gets more pronounced up the ladder of the hemiechine clade. These characteristics are unique within erinaceids, with the transformation series seemly directed and linear in behavior; one phenotype seems to transform into the next, directionally (state 0 → state 1→ state 2 → state 3 → state 4).

Alternative phylogenetic scenarios for *Mesechinus* and *Erinaceus* are recovered from the unweighted versus weighted analyses. The unweighted analysis finds *Mesechinus* paraphyletic and basal to the erinaceines. This topology is supported by a posteriori coding of missing dental characters. However, the relationship was not supported by bootstrap analysis and Bremer support was one (Figure 5a). The weighted analysis otherwise suggests *Mesechinus* is monophyletic, a hypothesis supported by four morphological characters (TS 11.0; 13.0; 31.1; 41.1). The first three are unique to all of the erinaceids, and the latter, is unique to erinaceines. These characters include the presence of a large robust jugal (as opposed to small, remedial or absent), an unfused lacrimal suture, a compressed suprameatal fossa, and a promontorium with a posteromedial wall and bullar roof formed mostly squamosal bone. These cranial characters show no plasticity within the erinaceids; therefore we reject the notion that *Mesechinus* is paraphyletic (based on morphological data).

All previous studies have found *Erinaceus* to be a monophyletic group, how they are related to one another remains uncertain [5], [17]. In both the unweighted and weighted analyses, we found no evidence for the monophyly of *Erinaceus,* nor did we find evidence to reject it.

### Molecular Data

DNA was extracted from twenty specimens and 3,218 mitochondrial bp were sequenced (Table 1). Of these, the sequences of *Mesechinus* and *Neotetracus* are novel data. Additional sequences were obtained from GenBank (Table 1). In total, twenty-nine specimens representing fourteen species and representatives of all ten erinaceids genera were sampled. Outgroup taxa included three species from the Soricidae, one from the Talpidae, and one from the Solenodontidae. Their sequences were downloaded from GenBank (Table 1). No premature stop codon was found within *CYT B* or *ND2* genes. Several insertion/deletion mutations (indels) were observed in *ND2*.

**Table 1:** Samples and sequences used in this study.

Family (subfamily)	Species (subspecies)	Collection code	Specimen code	Collecting site	12S	*CYT B*	*ND2*
Erinaceidae	*Echinosorex gymnura*	–	*Echinosorex gymnura 0*	–	AF348079*	AF348079*	AF348079*
(Galericinae)	*Hylomys parvus*	–	*Hylomys parvus 0a*	Sumatra	–	–	DQ630430*
		–	*Hylomys parvus 0b*	Sumatra	–	–	DQ630429*
		–	*Hylomys parvus 0c*	Sumatra	–	DQ630427* #	–
		–	*Hylomys parvus 0d*	Sumatra	–	AH009816* #	–
		–	*Hylomys parvus 0e*	Sumatra	–	AH009817* #	–
	*Hylomys suillus*	KIZ0611076	*Hylomys suillus 1*	Yunnan, China	HQ857485	HQ857523	HQ857504
		KIZ0611095	*Hylomys suillus 2*	Yunnan, China	HQ857486	HQ857524	HQ857505
		–	*Hylomys suillus 0a*	Malaysia	AM905042*	AM905042*	AM905042*
		–	*Hylomys suillus 0b*	Java	AM905041*	AM905041*	AM905041*
	*Hylomys s. dorsalis*	–	*Hylomys suillus dorsalis*	Borneo	–	AH009815* #	–
	*Hylomys s. maxi*	–	*Hylomys s. maxi 0a*	Malaya	–	AH009809* #	–
		–	*Hylomys s. maxi 0b*	Malaya	–	AH009810* #	–
		–	*Hylomys s. maxi 0c*	Sumatra	–	AH009811* #	–
		–	*Hylomys s. maxi 0d*	Malaya	–	AH009812* #	–
	*Hylomys s. microtinus*	–	*Hylomys suillus microtinus*	Vietnam	–	AH009808* #	–
	*Hylomys s. siamensis*	–	*Hylomys s. siamensis 0a*	Thailand	–	AH009805* #	–
		–	*Hylomys s. siamensis 0b*	Thailand	–	AH009806* #	–
		–	*Hylomys s. siamensis 0c*	Thailand	–	AH009807* #	–
	*Hylomys s. suillus*	–	*Hylomys s. suillus 0a*	Java	–	AH009813* #	–
		–	*Hylomys s. suillus 0b*	Java	–	AH009814* #	–
	*Neohylomys hainanensis*	YP22621	*Neohylomys hainanensis 1*	Hainan, China	HQ857496	HQ857534	HQ857515
		YP22624	*Neohylomys hainanensis 2*	Hainan, China	HQ857497	HQ857535	HQ857516
		YP22629	*Neohylomys hainanensis 3*	Hainan, China	HQ857498	HQ857536	HQ857517
	*Neotetracus sinensis*	KIZ0806027	*Neotetracus sinensis 1*	Yunnan, China	HQ857494	HQ857532	HQ857513
		KIZ0503272	*Neotetracus sinensis 2*	Yunnan, China	HQ857495	HQ857523	HQ857514
	*Podogymnura truei*	–	*Podogymnura truei 0*	–	AF434823*	AF434829*	–
Erinaceidae	*Atelerix albiventris*	–	*Atelerix albiventris 0*	–	M95109.1*	–	–
(Erinaceinae)	*Erinaceus amurensis*	KIZ0908002	*Erinaceus amurensis 1*	Liaoning, China	HQ857482	HQ857520	HQ857501
		KIZ080825	*Erinaceus amurensis 2*	Hubei, China	HQ857483	HQ857521	HQ857502
	*Erinaceus concolor*	–	*Erinaceus concolor 0*	Russia	AY012099.1*	–	AF481516*
	*Erinaceus europaeus*	–	*Erinaceus europaeus 0*	Sweden	NC 002080*	NC 002080*	NC 002080*
	*Hemiechinus auritus*	KCB88023	*Hemiechinus auritus 1*	–	HQ857484	HQ857522	HQ857503
		–	*Hemiechinus auritus 0*	–	NC 005033*	NC 005033*	NC 005033*
	*Mesechinus dauuricus*	KIZ0907004	*Mesechinus dauuricus 1*	Liaoning, China	HQ857487	HQ857525	HQ857510
		KIZ027004	*Mesechinus dauuricus 2*	Liaoning, China	HQ857488	HQ857526	HQ857509
		KIZ0910001	*Mesechinus dauuricus 3*	Ningxia, China	HQ857489	HQ857527	HQ857508
		KIZ027005	*Mesechinus dauuricus 4*	Ningxia, China	HQ857490	HQ857528	HQ857506
		KIZ027006	*Mesechinus dauuricus 5*	Ningxia, China	HQ857491	HQ857529	HQ857507
	*Mesechinus hughi*	KIZ027003	*Mesechinus hughi 1*	Shanxi, China	HQ857492	HQ857530	HQ857512
		KIZ027007	*Mesechinus hughi 2*	Shanxi, China	HQ857493	HQ857531	HQ857511
	*Paraechinus aethiopicus*	Qatar-S3	*Paraechinus aethiopicus 3*	Qatar	HQ857499	HQ857537	HQ857518
		Qatar-S4	*Paraechinus aethiopicus 4*	Qatar	HQ857500	HQ857538	HQ857519
Soricidae	*Crocidura russula*	–	*Crocidura russula 0*	Swiss	NC 006893*	NC 006893*	NC 006893*
	*Episoriculus fumidus*	–	*Episoriculus fumidus 0*	Taiwan, China	NC 003040*	NC 003040*	NC 003040*
	*Sorex unguiculatus*	–	*Sorex unguiculatus 0*	–	NC 005435*	NC 005435*	NC 005435*
Talpidae	*Talpa europaea*	–	*Talpa europaea 0*	Sweden	NC 002391*	NC 002391*	NC 002391*
Solenodontidae	*Solenodon paradoxus*	–	*Solenodon paradoxus 0*	–	AF076646*	AF434830*	–

* Sequences downloaded from GenBank.

# Only used for additional *CYT B* analysis of *Hylomys*.

**Figure 6:**
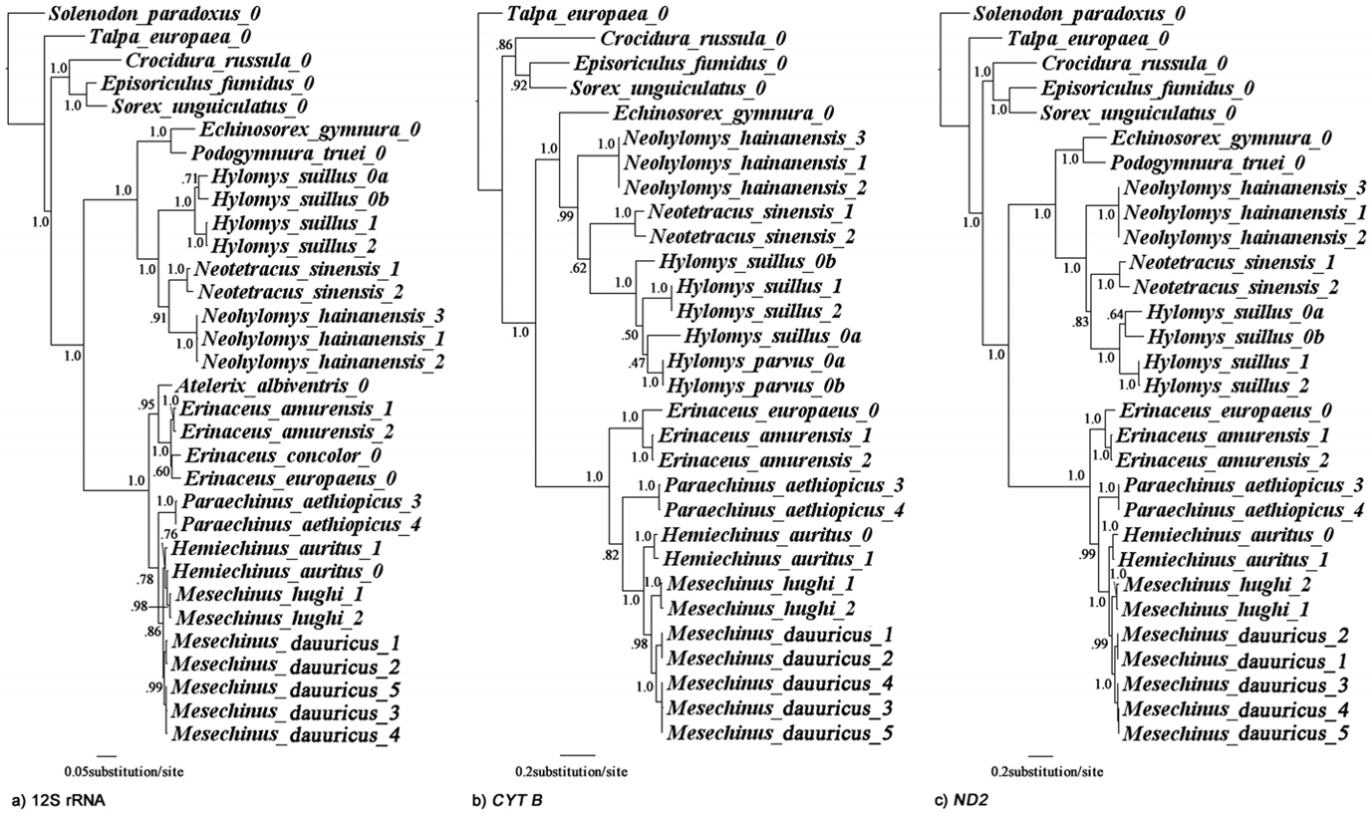
Mitochondrial phylogeny. Result of Bayesian phylogenetic analyses of three mitochondrial genes. Node numbers indicate Bayesian posterior probabilities.

The three partitioned analyses (12S rRNA; *CYT B*; *ND2*) discovered similar topologies with a few regions of incongruence (Figure 6a–c, respectively). While the 12S rRNA data discovered a sister taxon relationship between *Neotetracus* and *Neohylomys* (PP = 0.91, Figure 6a), the *CYT B* and *ND2* genes discovered no such relationship. The results of the latter two genes indicate that *Neohylomys* is basal to *Hylomys suillus* + *Neotetracus* (Figure 6b and c; PP = 0.62 and 0.83, respectively). Nonetheless, monophyly of *Hylomys* + *Neohylomys* + *Neotetracus* was consistently supported (PP≥0.99). The *CYT B* data discovered that *H. parvus* was more closely related to *H. suillus*, a result only found by the partitioned dental data set (Figure 5d and Figure S1d), a dataset known to be unreliable when considered in absence of other characters [15].

All the gene trees recovered the same hypothesis: *Mesechinus* and *Hemiechinus* are sister taxa. The partitioned morphological data however is much less conclusive (see Morphology discussion).

### Combined Data

Incongruencies across different data sets is not a novel observation within mammalian phylogenetic analyses [23], therefore, as a final test, we performed three combined analyses (genes and morphology + genes) with the expectation that the morphological data would improve branch support for relationships discovered by the genetic data [24], [25]. As in the previous analyses, we considered fourteen and twenty-two erinaceid taxa.

**Figure 7:**
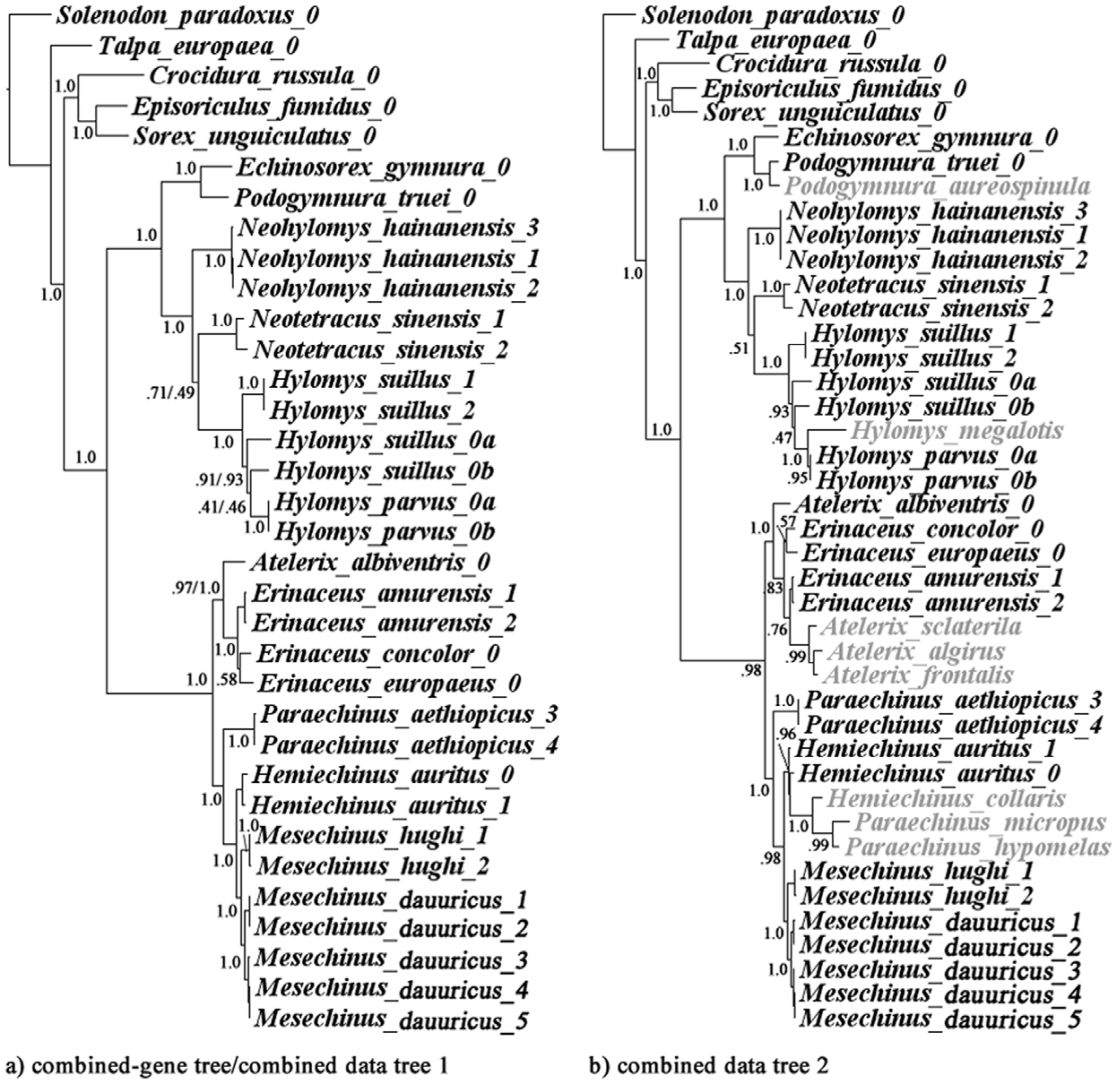
Combined-data phylogeny. Result of Bayesian phylogenetic analyses using combined genes or genes-morphology combined data set including 14 erinaceids species (a) and 22 species (b). Node numbers indicate Bayesian posterior probabilities. Only one value is presented if the posterior probabilities are identical. Taxa shaded in grey have no available gene sequences in this study.

The three-gene combined data and combined-data set 1 revealed the same topology and most of the interspecific relationships are strongly supported with few exceptions (Figure 7a). On the other hand, combined-data set 2 revealed a poorly supported tree (Figure 7b). Among the unsequenced taxa, the phylogenetic position of *P. aureospinula* and *H. megalotis* were strongly supported to be sister to their congeneric species (PP = 1.0). *Paraechinus hypomelas* + *P. micropus* are posited to be sister taxa to *Hemiechinus collaris* (PP = 0.96). *Atelerix*, *Erinaceus* and *Paraechinus* are all discovered to be paraphyletic. These results may be due to a posteriori assignment of missing data [26] and/or the inclusion of taxa with too few informative morphological characters [19], [20]. Interestingly, despite the low posterior probabilities, this tree is still congruent with the combined-data tree 1, suggesting it may act as a working hypothesis for these unsequenced taxa. To err on the side of caution, we will leave the examination of combined-data tree 2 to further studies and herein limit our discussion to the combined-data tree 1.

The combined-data tree 1 supports many relationships discovered in previous studies. The two subfamilies are strongly supported as reciprocal monophyletic groups. Within the subfamily Galericinae, the monophyly of (*Echinosorex+Podogymnura)*, and the *Hylomys* group (*Hylomys+Neotetracus+Neohylomys*) are well supported (PP = 1.0). Within the subfamily Erinaceinae, the monophyly of*Atelerix + Erinaceus,* and *Paraechinus* + [*Hemiechinus* + *Mesechinus*] are also strongly supported (PP = 1.0).

*Hylomys parvus* is embedded within *H. suillus* making the latter paraphyletic. *Mesechinus* is strongly supported as the sister taxon to *Hemiechinus auritus,* with *Paraechinus aethiopicus* basal to that clade. This topology challenges previous hypotheses of *Mesechinus* outside of the hemiechine clade (Figure 4b, c and h). Relationships discovered within *Erinaceus* were all poorly supported and characterized by extremely short branch lengths, an indication of rapid cladogenesis [27]. In contrast, branch lengths within Galericinae, are discovered to be quite long (especially for the *Hylomys* group), indicating more ancient origins than the spiny hedgehogs.

To summarize the overall results: relationships within the Erinaceinae were better resolved by the mitochondrial coding gene data (Figure 6b–c). In contrast, relationships within the Galericinae were better resolved by 12S rRNA (Figure 6a). The most strongly supported clades recovered in the combined-data set analyses were also recovered in all three partitioned gene analyses. The only one exception was the relationships within the *Hemiechinus* group (to include *Mesechinus* and *Paraechinus*). The sister taxon relationship of *Hemiechinus* and *Mesechinus* is strongly supported by the two coding genes but unresolved in the 12s rRNA gene tree, which may be attributed to insufficient phylogenetic signal.

## Discussion

The significant incongruence between the two data sets might be the result of adaptive evolution of phenotypic characters [28], maternal inheritance pathway of mitochondrial genes [29] or hybridization [30]. To better understand this question, it would be necessary to address broader taxon sampling, multiple genes to reconstruct a robust species tree to identify the source of error [31], and review of more specimens to address the missing data problems. In this paper, we consider the combined-data tree 1 to be the strongest supported hypothesis posited thus far.

Though we have confirmed many relationships proposed in previous analyses, the novel findings are: (i) paraphyly of *H. suillus,* (ii) deep divergence within the *Hylomys* groups (*Hylomys*, *Neohylomys* and *Neotetracus*), and (iii) novel relationships of the *Hemiechinus* group (*Paraechinus* + [*Hemiechinus* + *Mesechinus]*).

**Figure 8:**
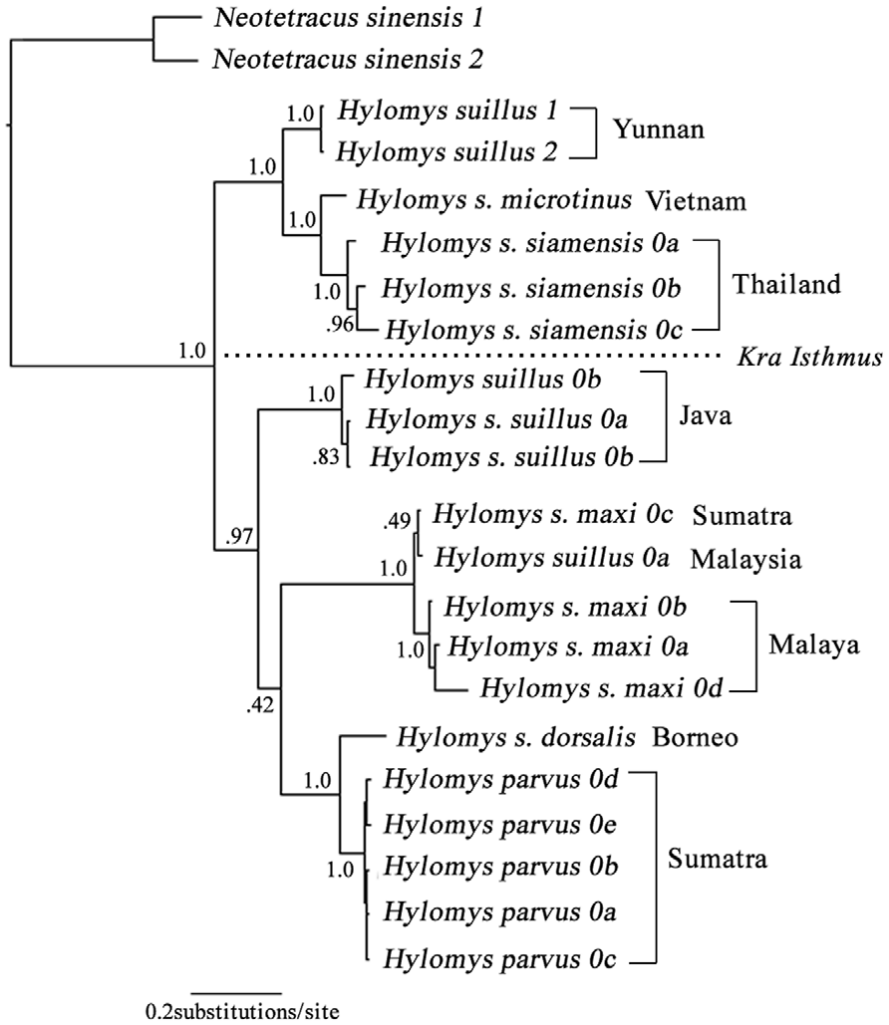
*Hylomys* phylogeny based on partial *CYT B*. Result of Bayesian phylogenetic analyses of *Hylomys* based on partial *CYT B* genes. Node numbers indicate Bayesian posterior probabilities. Distribution of the two major clades was divided by the Kra Isthmus.

The paraphyly of *H. suillus* and the strikingly large genetic distance within *H. suillus* have already been demonstrated [16], [32]. According to our results, the Kimura 2-parameter (K2P) distance of complete *CYT B* within *H. parvus/suillus* complex ranged from 0.0% – 20.3% which is higher than the average genetic distance for sister species in mammals (8.1%) [33]. Furthermore, in an extended analysis using additional partial *CYT B* sequences, all specimens of *H. parvus/suillus* fell into two strongly supported monophyletic clades (Figure 8; PP≥0.97), one of which is distributed throughout Indochina, while the other is limited to Malaya and the Sunda Islands. Presumably, the Kra Isthmus acted as a geographic barrier between the two clades. The inconsistency between taxonomic designations, large genetic distances and the strong geographic patterns imply that the subspecies within *Hylomys suillus* may deserve full species status as suggested by Ruedi and Fumagalli [16]. As mentioned in the Results section, the morphological data for *H. parvus* was gleaned from the literature; consequently there are seventy-one missing characters for this taxon. These missing data should be rectified for future study and more specimens of each subspecies of *H. suillus* should be included.

In previous hypotheses, the species nomenclature within *Hylomys* (e.g. *Neohylomys* and *Neotetracus*) seems to be author dependent [1], [13], [17]. In this study, the combined-data set could not resolve the relationships among the three. Nonetheless, the three taxa are characterized by deep divergences. K2P distances between the genera are as high as11.6%–16.1% (12S); 22.1%–26.8% (*CYT B*) and 30.9%–33.7% (*ND2*). The K2P distances between *Crocidura russula* (NC_006893; Crocidurinae) and *Sorex unguiculatus* (NC_005435; Soricinae) in two different shrew subfamilies are 14.6% (12S), 23.7% (*CYT B*) and 35.1% (*ND2*). The strikingly large genetic distances within the *Hylomys* group indicate ancient (maybe also rapid) divergence events, which coincides with the early Miocene fossil records (MN4) [34].

Interestingly, our morphological analyses could not resolve their relationships either. The data indicate that there is considerable support for the monophyly of *Hylomys suillus* + (*Neotetracus + Neohylomys*). There are four robust characters that support it: (TS 6.1; 14.1; 16.1; 21.1), which are listed in the morphological relationships section. The sister taxon relationship of *N. sinensis* and *N. hainanensis* has been discovered in several pervious analyses [1], [5], [12] on the basis of their shared loss of P1 or their loss of p1, depending on how the transformation was cast along the long branches. Frost et al. [17] found *H. suillus* and *N. sinensis* to be sister taxa; their hypothesis was supported by the reappearance of P1, which they rejected as an artifact of a posteriori coding. We discovered no concrete evidence however for a sister taxon relationship between *Neotetracus* and *Neohylomys* because both characters in questions (P1 and p1) cannot be optimized on the tree due to plasticity in closely related taxa [15], [35], [36]. A better understanding of some of these commonly used morphological characters is needed.

The most significant incongruence within the Erinaceinae is the phylogenetic positions of *Mesechinus* and *Paraechinus.* The molecular evidence suggests that *Mesechinus* is the sister taxon to *Hemiechinus,* as indicated by Corbet [1]. However, the morphological tree strongly favors the hypothesis that the *Paraechinus* is sister to *Hemiechinus* (Figure 5 and Figure S1), which is strongly supported by the inflation of the entire auditory region, a characteristic unique among all erinaceids (see Morphology Results). The inflation of the skull in and around the auditory region is indicative of arid to semi-arid dwelling mammals, who must hone in on the low frequency sounds of the wing beats of their principal predators–raptors and is exhibited in other small to medium sized arid-dwelling mammals (e.g., [37], [38])**.** The inflation of bullae in both *Hemiechinus* and *Paraechinus* alternatively, could be the result of convergent adaptation to an arid environment. This hypothesis however, warrants more consideration because this morphological transformation series is unique for erinaceids. Further genetic studies could test the relationships among these taxa. If convergent evolution is indeed a viable hypothesis, more morphological characters should be reexamined to ascertain the subtle differences in the bullar inflation.

In this study, we obtained sequences of all erinaceids from China including *Mesechinus* and *Neotetracus* for the first time. We not only confirmed many hypotheses proposed by previous authors, including the monophyly of both subfamilies, the *Hylomys* group, but also found deep genetic divergence within Galericinae. Novel relationships between *Hemiechinus*, *Mesechinus* and *Paraechinus*, as well as incongruencies between genes and morphological data concerning the phylogenetic positions of *Hylomys parvus* and *Mesechinus* and between *Hemiechinus* and *Paraechinus* were recovered. These results indicate that there are non-negligible issues with regard to species nomenclature, especially within the *Hylomys* group, which have been shown to have great depth in their genetic diversity. Even so, the lack of complete taxon sampling or multiple unlinked genes prevent us from identifying robust relationships among particular taxa.

A more comprehensive sampling of species, to include representatives of *Hylomys megalotis*, *Hemiechinus* spp. and *Paraechinus* spp. is necessary to test the genetic relationships found herein. The morphological characters also need to be reexamined. With a more robust tree of extant species, we hope to recover the source of incongruencies between genes and morphological characters and provide solid evidence for more appropriate nomenclature. Only after that, the morphological data matrix will be confidently applied to test the phylogenetic positions of fossil species as well as biogeography and timing of diversification of the family.

## Materials and Methods

### Ethics Statement

The capture, handling, and care of mammals followed the guidelines approved by the American Society of Mammalogists [39]. All animal samples from China were obtained following the regulations for the implementation of China on the protection of terrestrial wild animals (State Council Decree [1992] No.13) and approved by the Ethics Committee of Kunming Institute of Zoology, Chinese Academy of Sciences, China (no specific permit number). The two samples from Qatar were ear clipping from dead body found by highways, and did not need permit in this case.

### Taxon Sampling

Twenty-two out of the twenty-four named erinaceid species were included in morphological analyses. *Mesechinus hughi* is a newly included species; it was recovered from the Qinling Mountain in China in 2009 (Table 1) and was reviewed herein by author Gould. We sampled eight erinaceid species within Erinaceidae, including three from Galericinae and five from Erinaceinae for genetic study. All of the known species within China were sampled. All nomenclature in this paper follows Hutterer [40]. Shrews, moles and solenodons were selected as outgroups (Table 1) for phylogenetic studies because they are the closest relatives of erinaceids [8], [41], [42].

### Morphological Characters

Phylogenetic relationships based on morphological characters have been independently corroborated several times [1], [5], [13], [15], [17], [21]. The majority of the characters analyzed in this study have been elaborated on extensively by Frost et al., [17], Gould [5], and Gould’s dissertation [21]. A total of one hundred and thirty-five characters were compiled in the morphological data matrix, including eight new characters (Text S1). Of these characters, sixty-one are cranial, fifty-nine are dental, eight are postcranial, and seven are pelage. Not all specimens could be personally reviewed; hence we relied upon the literature to score the morphological characters for *Hylomys parvus* and *H. megalotis*. The character states for these species came from Jenkins and Robinson [13]. Twenty-three dental characters were not coded for most extant species, so these transformation series were subsequently omitted from the analyses. They are still listed in Table S1 for posterity reasons, but are denoted in *italics* in Text S1.

### DNA Characters

All samples from China were derived from liver or muscle tissues, which were stored in ethanol at −70°C. Two roadkill hedgehogs were found along highways in Qatar from which ear clipping were taken. The DNA was extracted using the phenol/proteinase K/sodium dodecyl sulphate method [43]. Three mitochondrial gene regions including 12S rRNA [962–982 bp], Cytochrome B (*CYT B*) [1,140 bp] and NADH dehydrogenase subunit 2 (*ND2*) [1,044 – 1,047 bp] were amplified with rTaq DNA polymerase (Takara, Dalian, China). Primers used are provided in Table 2.

**Table 2:** Primers used in PCR reaction and sequencing.

Locus	Primer Name	Primer Sequences (5′-3′)	sense/anti-sense	Cited Source
12S	EML4	GGACTGAAGCAAAGCACTGAAAATG	sense	This study
	EMH4	ATCACCAGACTCGTTAGGCTTTTCAC	anti-sense	This study
*ND2*	ERL4	AGGTAGGCTAAACAAGCTATCGGGC	sense	This study
	ERH4	CTTAACGCTTTGAAGGCTTTTGGTC	anti-sense	This study
*CYT B* *	EDL6	CCCTAAGGATATGAAAAACCATCGTT	sense	This study
	EDH6	GGTTTCCCATCTTTGGTTTACAAGAC	anti-sense	This study
	L14724_hk4	CCCGTGATATGAAAAATCATTGTTG	sense	This study
	H15915_hk4	CCGTTCTCTTCTCTGGTTTACAAAAC	anti-sense	This study
	H15427	ATGTCAACTTTGGGTGTTGATGGT	anti-sense	This study
	L14724_hk3	GGACTTATGACATGAAAAATCATCGTTG	sense	[27]
	H15443_hk1	GAATACCAGCTTTGGGTGTTGATG	anti-sense	This study

* EDL6 and EDH6 were used for *Hylomys* and *Neotetracus*. L14724_hk3 and H15443_hk1 were used for *Neohylomys*. L14724_hk4 and H15427 were used for *Mesechinus dauuricus*. L14724_hk4 and H15915_hk4 were used for the other species.

A universal touchdown PCR program [44] consisting of two phases was used. Phase 1 included an initial step of 94°C for three minutes, followed by ten cycles of 92°C for 60 s, annealing for 60 s, and 72°C for 60 s. The annealing temperature was decreased by 0.5°C per circle from 55°C to 50.5°C. Phase 2 consisted of twenty-five cycles of 92°C for 60 s, 50°C for 60 s, and 72°C for 60 s and followed by the final extension at 72°C for 10 min. All PCR products were purified using UNIQ-10 spin column DNA gel extraction kit (Sangon, Shanghai, China). Purified products were directly sequenced with PCR primers using the BigDye Terminator Cycle kit v3.1 on ABI 3730xl sequencer in Tiangen Biotech Co, LTD., in Beijing.

Nucleotide sequences were edited using SeqMan and EditSeq in DNASTAR package v7.1 (DNASTAR, Inc., USA) and aligned with ClustalX v1.83 [45]. Coding genes were translated to amino acids following the identification of any premature stop codon. Additional sequences downloaded from GenBank were added to alignments. *CYT B* and *ND2* were aligned using amino acid sequences which allow identification of insertion/deletion (indel) polymorphisms. Alignment of 12S rRNA was further modified based on secondary structure following Springer and Douzery [46]. Stem [504 bp] and loop regions [ca. 524 bp] were recognized. Alignment of the loop region was then submitted to BMGE (http://mobyle.pasteur.fr/), and highly variable/uncertain regions were removed automatically using default setting [47]. Ca. 362 bp alignment of loop regions were obtained for phylogenetic analyses.

### Phylogenetic Analyses

We used maximum parsimony (MP) to analyze the morphological data, and employed Bayesian probabilities on both the molecular and combined-data sets. MP analyses were implemented using PAUP 4.0b10 [48]. We performed heuristic searches with 1000 random addition replicates using the TBR branch-swapping algorithm and collapse all zero length branches (collapse  =  minbrlen). The characters were optimized using “accelerated transformation” on the trees in memory (opt  =  acctran). MP bootstrap values were calculated on 1000 replicates of random addition sequence. All morphological characters were first weighted equally. Gould suggested the dental variation is intemperate both inter- and intra-specifically within the Erinaceidae and the phylogenetic resolving power of the dental data is contingent on the inclusion of other data, i.e., cranial, postcranial and pelage [15]. Thus, we performed additional analyses: (i) using only non-dental characters (#76), (ii) using only dental characters (#36) and (iii) weighted the dental characters vs. non-dental  = 1∶3 against homoplasy [22]. Weighted characters were treated as repeat counts during bootstrap (wts = repeatcnt). Two morphological data sets were analyzed. The first is the data matrix containing all twenty-two ingroup species we have morphological data for, and the second contains the fourteen species for which we have genetic data. We performed all four analyses for both data matrixes. When analyzed the matrixes using only dental characters for 23 taxa, the maximum number of trees that can be saved was set to 10,000. Tree lengths were calculated with MacClade 4 [49]. Bremer supports were also calculated using TreeRot v3 [50]. Apomorphy lists for all morphological trees and combined tree 1 (see below) were generated by PAUP and provided as Text S2.

Bayesian analyses were conducted on each of the genes, three-gene combined data and two morphology + genes combined-data sets with MrBayes v3.1.2 [51] via the CIPRES Portal v2.2 [52]. For the combined-data sets, first, for the fourteen species those have at least one gene were included (three-gene and combined-data set 1); second, all of the twenty-two living species including those without gene sequences (combined-data set 2). The model of DNA evolution was determined by Bayesian Information Criterion (BIC) [53] in jModelTest v0.1.1 [54], [55] for stem and loop regions of 12S and each codon position of *ND2* and *CYT B* separately [56]. BIC was chosen because of its high accuracy and precision [57]. In the model test, likelihood calculations were carried out with Phyml [54]. Three substitution schemes (JC, HKY and GTR) were selected, and a proportion of invariant sites were not included in the model selection following Meredith et al. [8]. For each model, a ML tree was estimated to optimize the topology for tree length and parameter estimation (ML optimized). Substitution models for all partitions are provided in Table S2. For morphological characters, we used the default ?Mk? model [58] and set?coding  =  variable? and ?rates  =  gamma? [25], [59]. The ordering of morphological characters was inconsistent with the MP analyses. The monophyly of Eulipotyphlan and Erinaceidae + Soricidae were constrained according to Roca et al. [42]. We performed a MCMC search of ten million generations, using four chains, two independent runs, and sampling every 1000 generations. Parameters between partitions were unlinked [unlink statefreq = (all) revmat = (all) shape = (all)]. Partition-specific rates were invoked [prset applyto =  (all) ratepr = variable]. All analyses were repeated four times. Tracer v1.5 was used to make sure all analyses reach the same posterior and estimated the convergences by calculating effective sample sizes (ESSs) [60]. ESSs for all parameters were higher than 1,000 after 3 million generations, so the first 30% of the generations were discarded as burn-in. All four analyses were combined to summarize the final tree and branch lengths. According to Huelsenbeck and Rannala [61], posterior probabilities (PP) ≥0.95 are considered statistically (i.e., “strongly”) supported.

An additional Bayesian analysis focused on the *Hylomys parvus*/*suillus* complex was performed. Partial *CYT B* sequences (539 bp) from Ruedi and Fumagalli [16]’s research were download from GenBank (access nos: AH009805-AH009817) in combination with other *CYT B* sequences of these two species. The DNA evolutionary models for the 1^st^, 2^nd^ and 3^rd^ codon were SYM+G, HKY+G and HKY, respectively.

In total, we performed fifteen analyses to ascertain the phylogenetic signals of the character data sets (differing genes, morphological, dental vs. non-dental) and the taxa (not all taxa had genetic data) (Table 3).

**Table 3:** Phylogenetic analyses performed in this study.

Analyses	Characters (number of characters/base pairs used in phylogenetic analyses)	Taxa (number of taxa/sequences)	Method	Weight strategy inMP analyses	Figure
Morphological analyses	Adjusted characters (#112)	22 species plus 1 outgroup (#23)	MP	Equal weight	Figure 5a
	Adjusted characters (#112)	22 species plus 1 outgroup (#23)	MP	non-dental charactersup-weighted	Figure 5b
	Non-dental (#76)	22 species plus 1 outgroup (#23)	MP	Equal weight	Figure 5c
	Dental characters (#36)	22 species plus 1 outgroup (#23)	MP	Equal weight	Figure 5d
	Adjusted characters (#112)	14 species plus 1 outgroup (#15)	MP	Equal weight	Figure S1a
	Adjusted characters (#112)	14 species plus 1 outgroup (#15)	MP	non-dental charactersup-weighted	Figure S1b
	Non-dental (#76)	14 species plus 1 outgroup (#15)	MP	Equal weight	Figure S1c
	Dental characters (#36)	14 species plus 1 outgroup (#15)	MP	Equal weight	Figure S1d
Molecular analyses	12S (504 bp stem and 362 bp loop region)	13 species plus 5 outgroup (# 32)	Bayesian	N.A.	Figure 6a
	*CYT B* (1,140 bp)	11 species plus 4 outgroup (# 30)	Bayesian		Figure 6b
	*ND2* (1,053 bp)	11 species plus 4 outgroup (# 30)	Bayesian		Figure 6c
Combined analyses	12S + *CYT B* +*ND2* (3,059 bp)	14 species plus 5 outgroup (# 30)	Bayesian		Figure 7a
	Adjusted characters (#112) +3 genes (3,059 bp)	14 species plus 5 outgroup (# 34)	Bayesian		
		22 species (8 have no genetic data)plus 5 outgroup (# 34)	Bayesian		Figure 7b
Additional analyses	Partial *CYT B* (539–1,140 bp)	*Hylomys suillus/parvus* plus 3outgroups (# 23)	Bayesian		Figure 8

The NEXUS files for MP and Bayesian analyses are available as supplementary information (ZIP S1).

## Supporting Information

Figure S1 **Morphological phylogeny of 14 erinaceids.** Morphological strict consensus trees for 14 species using equal weighted (a) and unequal weighted (b), non-dental (c) and dental-only characters (d). Numbers above branches indicate bootstrap values, those below the branches indicate Bremer supports.
(TIF)

Table S1 **Morphological data matrix.** Morphological data matrix for the 22 erinaceid species and the outgroup soricoid.
(TXT)

Table S2 **DNA substitution models.** DNA substitution models and MrBayes setting of the 12S rRNA and each codon of the two coding genes.
(DOC)

Text S1 **Morphological Transformation Series.** Morphological Transformation Series, All characters are polarized and ordered unless otherwise specified.
(DOC)

Text S2 **Tree information and apomorphy lists.** Tree information and apomorphy list for each morphological tree and the combined tree 1.
(DOC)

ZIP S1 NEXUS files used for morphological (PAUP), genetic (MrBayes) and morphology-genes combined (MrBayes) analyses.
(ZIP)
