# Supplementary figures and images for: An Estimation of Erinaceidae Phylogeny: A Combined Analysis Approach
Source: PLoS One. 2012 Jun 20;7(6):e39304. doi: 10.1371/journal.pone.0039304 (PMC3380021; doi:10.1371/journal.pone.0039304)

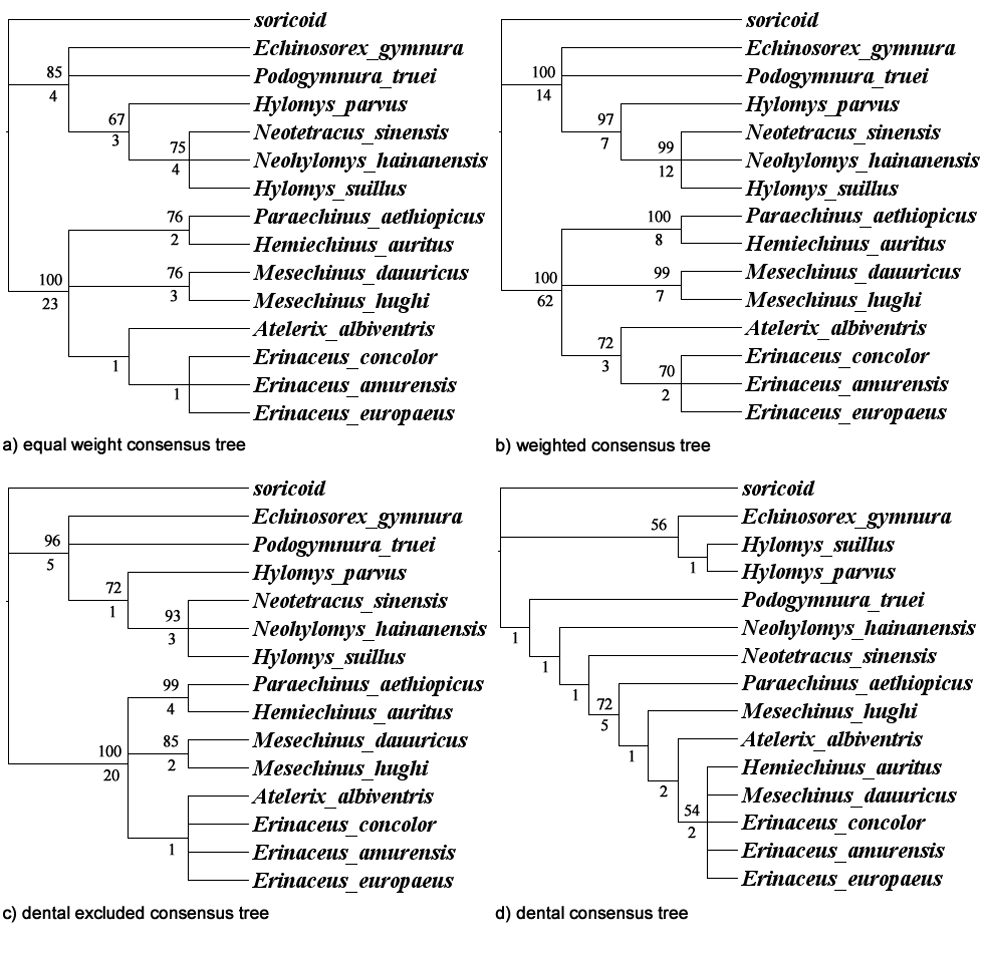

Supplement: Figure S1 — Morphological phylogeny of 14 erinaceids. Morphological strict consensus trees for 14 species using equal weighted (a) and unequal weighted (b), non-dental (c) and dental-only characters (d). Numbers above branches indicate bootstrap values, those below the branches indicate Bremer supports. (TIF) [file pone.0039304.s001.tif]
