# Supplementary material for: An Estimation of Erinaceidae Phylogeny: A Combined Analysis Approach
Source: PLoS One. 2012 Jun 20;7(6):e39304. doi: 10.1371/journal.pone.0039304 (PMC3380021; doi:10.1371/journal.pone.0039304)
Supplement: Table S2 — DNA substitution models. DNA substitution models and MrBayes setting of the 12S rRNA and each codon of the two coding genes. (DOC) [file pone.0039304.s003.doc]

**Table S2. DNA evolutionary model selected.**

| Partition | Model | MrBayes setting and Prior |
| --- | --- | --- |
| 12S stem | SYM+G | nst=6 rates=gamma, statefreqpr=fixed(equal) |
| 12S loop | GTR+G | nst=6 rates=gamma |
| *CYT* *B* 1st | GTR+G | nst=6 rates=gamma |
| *CYT* *B* 2nd | HKY+G | nst=2 rates= gamma |
| *CYT* *B* 3rd | HKY+G | nst=2 rates= gamma |
| *ND2* 1st | GTR+G | nst=6 rates=gamma |
| *ND2* 2nd | HKY+G | nst=2 rates=gamma |
| *ND2* 3rd | HKY+G | nst=2 rates=gamma |
