# Supplementary material for: An Estimation of Erinaceidae Phylogeny: A Combined Analysis Approach
Source: PLoS One. 2012 Jun 20;7(6):e39304. doi: 10.1371/journal.pone.0039304 (PMC3380021; doi:10.1371/journal.pone.0039304)
Supplement: Text S2 — Tree information and apomorphy lists. Tree information and apomorphy list for each morphological tree and the combined tree 1. (DOC) [file pone.0039304.s005.doc]

**Test S2. Tree information and** **Apomorphy lists.**

**1. Strict consensus morphological tree of 23 taxon using equal weighted characters.**

Tree length = 207, Consistency index (CI) = 0.5652, Homoplasy index (HI) = 0.4348, CI excluding uninformative characters = 0.5408, HI excluding uninformative characters = 0.4592, Retention index (RI) = 0.7982, Rescaled consistency index (RC) = 0.4512


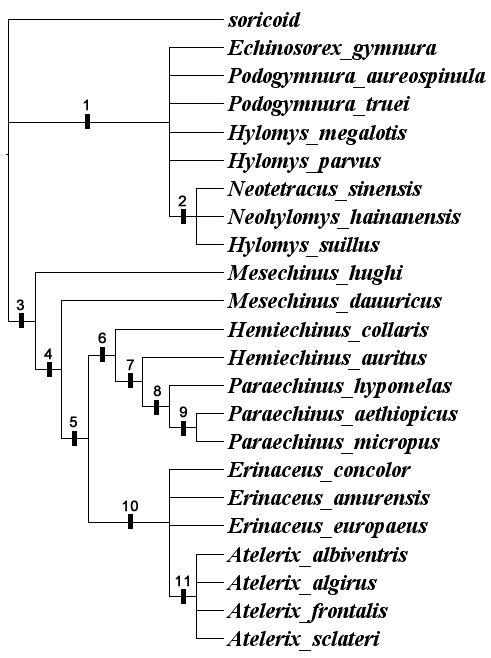


**Apomorphy lists:**

| Stem | Character | Steps | CI | Change |
| --- | --- | --- | --- | --- |
| Stem 1 | 8 | 1 | 0.500 | 0 ==> 1 |
|  | 9 | 1 | 0.750 | 2 ==> 1 |
|  | 11 | 1 | 1.000 | 0 --> 2 |
|  | 13 | 1 | 0.500 | 0 ==> 1 |
|  | 22 | 1 | 0.250 | 0 --> 1 |
|  | 25 | 1 | 0.200 | 0 ==> 1 |
|  | 42 | 1 | 0.333 | 0 ==> 1 |
|  | 59 | 1 | 0.333 | 0 ==> 1 |
|  | 121 | 1 | 1.000 | 0 ==> 1 |
|  | 123 | 1 | 1.000 | 0 ==> 1 |
|  | 124 | 1 | 1.000 | 0 ==> 1 |
|  | 125 | 1 | 1.000 | 0 ==> 1 |
|  | 126 | 1 | 1.000 | 0 ==> 1 |
| *Echinosorex gymnura* | 5 | 1 | 0.333 | 0 --> 1 |
|  | 7 | 1 | 0.500 | 0 ==> 1 |
|  | 8 | 1 | 0.500 | 1 ==> 0 |
|  | 9 | 1 | 0.750 | 1 --> 0 |
|  | 10 | 1 | 0.333 | 1 ==> 2 |
|  | 21 | 1 | 0.286 | 1 ==> 0 |
|  | 22 | 1 | 0.250 | 1 --> 0 |
|  | 25 | 1 | 0.200 | 1 ==> 0 |
|  | 26 | 1 | 1.000 | 0 ==> 1 |
|  | 35 | 1 | 0.500 | 1 ==> 0 |
|  | 42 | 1 | 0.333 | 1 ==> 0 |
|  | 59 | 1 | 0.333 | 1 ==> 0 |
|  | 61 | 1 | 0.333 | 0 ==> 1 |
|  | 64 | 1 | 0.500 | 1 ==> 0 |
|  | 70 | 1 | 0.250 | 1 --> 0 |
|  | 82 | 1 | 0.167 | 1 ==> 0 |
|  | 86 | 1 | 0.500 | 1 --> 0 |
|  | 112 | 1 | 0.333 | 0 ==> 1 |
|  | 122 | 1 | 0.500 | 0 --> 1 |
|  | 135 | 1 | 0.333 | 0 --> 1 |
| *Podogymnura aureospinula* | 5 | 1 | 0.333 | 0 --> 1 |
|  | 9 | 1 | 0.750 | 1 --> 0 |
|  | 10 | 1 | 0.333 | 1 ==> 0 |
|  | 42 | 1 | 0.333 | 1 ==> 0 |
|  | 59 | 1 | 0.333 | 1 ==> 0 |
|  | 61 | 1 | 0.333 | 0 ==> 1 |
|  | 63 | 1 | 0.400 | 0 ==> 1 |
|  | 66 | 1 | 0.333 | 0 ==> 1 |
|  | 135 | 1 | 0.333 | 0 --> 1 |
| *Podogymnura truei* | 5 | 1 | 0.333 | 0 --> 1 |
|  | 9 | 1 | 0.750 | 1 --> 0 |
|  | 10 | 1 | 0.333 | 1 ==> 0 |
|  | 61 | 1 | 0.333 | 0 ==> 1 |
|  | 63 | 1 | 0.400 | 0 ==> 1 |
|  | 66 | 1 | 0.333 | 0 ==> 1 |
|  | 135 | 1 | 0.333 | 0 --> 1 |
| Stem 2 | 4 | 1 | 0.500 | 0 ==> 1 |
|  | 6 | 1 | 0.500 | 0 ==> 1 |
|  | 14 | 1 | 1.000 | 0 ==> 1 |
|  | 15 | 1 | 0.500 | 1 ==> 0 |
|  | 16 | 1 | 1.000 | 0 ==> 1 |
|  | 19 | 1 | 0.333 | 0 ==> 1 |
|  | 22 | 1 | 0.250 | 1 --> 0 |
|  | 25 | 1 | 0.200 | 1 --> 0 |
|  | 74 | 1 | 0.667 | 0 ==> 1 |
|  | 83 | 1 | 0.333 | 0 ==> 1 |
|  | 92 | 1 | 0.500 | 2 ==> 0 |
|  | 122 | 1 | 0.500 | 0 --> 1 |
| *Neotetracus sinensis* | 1 | 1 | 0.333 | 0 ==> 1 |
|  | 25 | 1 | 0.200 | 0 --> 1 |
|  | 91 | 1 | 0.250 | 0 ==> 1 |
|  | 98 | 1 | 0.500 | 1 ==> 2 |
|  | 100 | 1 | 0.500 | 1 ==> 0 |
| *Neohylomys hainanensis* | 63 | 1 | 0.400 | 0 ==> 1 |
|  | 70 | 1 | 0.250 | 1 --> 0 |
| *Hylomys suillus* | 71 | 1 | 0.250 | 1 ==> 0 |
|  | 86 | 1 | 0.500 | 1 --> 0 |
|  | 88 | 1 | 0.333 | 0 ==> 1 |
|  | 109 | 1 | 0.500 | 0 ==> 1 |
| *Hylomys megalotis* | 1 | 1 | 0.333 | 0 ==> 1 |
|  | 5 | 1 | 0.333 | 0 --> 1 |
|  | 10 | 1 | 0.333 | 1 ==> 2 |
|  | 15 | 1 | 0.500 | 1 ==> 0 |
|  | 17 | 1 | 0.500 | 0 ==> 1 |
|  | 19 | 1 | 0.333 | 0 ==> 1 |
|  | 21 | 1 | 0.286 | 1 ==> 0 |
|  | 70 | 1 | 0.250 | 1 --> 0 |
|  | 71 | 1 | 0.250 | 1 ==> 0 |
|  | 82 | 1 | 0.167 | 1 ==> 0 |
| *Hylomys parvus* | 4 | 1 | 0.500 | 0 ==> 1 |
|  | 19 | 1 | 0.333 | 0 ==> 1 |
|  | 21 | 1 | 0.286 | 1 ==> 0 |
|  | 70 | 1 | 0.250 | 1 --> 0 |
|  | 71 | 1 | 0.250 | 1 ==> 0 |
|  | 83 | 1 | 0.333 | 0 ==> 1 |
| Stem 3 | 1 | 1 | 0.333 | 0 ==> 1 |
|  | 2 | 1 | 1.000 | 0 ==> 1 |
|  | 4 | 1 | 0.500 | 0 ==> 2 |
|  | 6 | 1 | 0.500 | 0 ==> 1 |
|  | 7 | 1 | 0.500 | 0 ==> 1 |
|  | 10 | 1 | 0.333 | 1 ==> 2 |
|  | 17 | 1 | 0.500 | 0 ==> 1 |
|  | 18 | 1 | 1.000 | 0 ==> 1 |
|  | 21 | 1 | 0.286 | 1 ==> 2 |
|  | 22 | 1 | 0.250 | 0 --> 1 |
|  | 24 | 1 | 1.000 | 0 ==> 1 |
|  | 27 | 1 | 1.000 | 0 ==> 1 |
|  | 28 | 1 | 1.000 | 0 ==> 1 |
|  | 30 | 1 | 1.000 | 0 ==> 1 |
|  | 31 | 1 | 0.500 | 0 --> 1 |
|  | 33 | 1 | 1.000 | 0 ==> 1 |
|  | 36 | 1 | 1.000 | 0 --> 2 |
|  | 40 | 1 | 1.000 | 0 ==> 1 |
|  | 46 | 1 | 1.000 | 0 ==> 1 |
|  | 47 | 1 | 1.000 | 0 ==> 1 |
|  | 58 | 1 | 1.000 | 0 ==> 1 |
|  | 60 | 1 | 1.000 | 0 ==> 1 |
|  | 63 | 1 | 0.400 | 0 --> 2 |
|  | 64 | 1 | 0.500 | 1 ==> 0 |
|  | 66 | 1 | 0.333 | 0 --> 1 |
|  | 74 | 1 | 0.667 | 0 ==> 2 |
|  | 83 | 1 | 0.333 | 0 ==> 1 |
|  | 92 | 1 | 0.500 | 2 --> 3 |
|  | 95 | 1 | 1.000 | 0 --> 1 |
|  | 114 | 1 | 1.000 | 0 ==> 1 |
|  | 128 | 1 | 0.500 | 0 --> 1 |
|  | 129 | 1 | 1.000 | 0 --> 1 |
|  | 131 | 1 | 1.000 | 0 --> 1 |
|  | 132 | 1 | 1.000 | 0 --> 1 |
|  | 133 | 1 | 1.000 | 0 --> 3 |
|  | 134 | 1 | 0.500 | 0 --> 1 |
| Stem 4 | 23 | 1 | 0.333 | 0 ==> 1 |
|  | 82 | 1 | 0.167 | 1 ==> 0 |
|  | 91 | 1 | 0.250 | 0 --> 1 |
| Stem 5 | 11 | 1 | 1.000 | 0 ==> 1 |
|  | 13 | 1 | 0.500 | 0 ==> 1 |
|  | 31 | 1 | 0.500 | 1 --> 0 |
|  | 41 | 1 | 0.500 | 1 ==> 0 |
| Stem 6 | 3 | 1 | 1.000 | 0 ==> 1 |
|  | 30 | 1 | 1.000 | 1 ==> 2 |
|  | 34 | 1 | 1.000 | 0 ==> 1 |
|  | 127 | 1 | 1.000 | 0 ==> 1 |
|  | 130 | 1 | 1.000 | 0 --> 2 |
|  | 131 | 1 | 1.000 | 1 --> 3 |
|  | 134 | 1 | 0.500 | 1 --> 0 |
| Stem 7 | 23 | 1 | 0.333 | 1 ==> 0 |
| Stem 8 | 12 | 1 | 1.000 | 1 ==> 2 |
|  | 30 | 1 | 1.000 | 2 ==> 3 |
|  | 43 | 1 | 1.000 | 0 ==> 1 |
|  | 44 | 1 | 1.000 | 0 ==> 1 |
|  | 133 | 1 | 1.000 | 3 ==> 2 |
| Stem 9 | 30 | 1 | 1.000 | 3 ==> 4 |
|  | 82 | 1 | 0.167 | 0 ==> 1 |
|  | 91 | 1 | 0.250 | 1 --> 0 |
|  | 92 | 1 | 0.500 | 3 --> 0 |
|  | 100 | 1 | 0.500 | 1 --> 0 |
|  | 109 | 1 | 0.500 | 0 --> 1 |
| *Paraechinus aethiopicus* | 12 | 1 | 1.000 | 2 ==> 3 |
| *Paraechinus micropus* | 22 | 1 | 0.250 | 1 ==> 0 |
|  | 23 | 1 | 0.333 | 0 ==> 1 |
| *Paraechinus hypomelas* | 88 | 1 | 0.333 | 0 ==> 1 |
| *Hemiechinus auritus* | 93 | 1 | 0.333 | 1 ==> 0 |
| Stem 10 | 32 | 1 | 0.500 | 0 ==> 1 |
|  | 110 | 1 | 0.333 | 1 --> 0 |
|  | 112 | 1 | 0.333 | 0 --> 1 |
|  | 128 | 1 | 0.500 | 1 --> 0 |
|  | 133 | 1 | 1.000 | 3 ==> 1 |
| Stem 11 | 25 | 1 | 0.200 | 0 ==> 1 |
|  | 29 | 1 | 1.000 | 0 ==> 1 |
|  | 73 | 1 | 1.000 | 1 ==> 0 |
|  | 92 | 1 | 0.500 | 3 --> 2 |
|  | 128 | 1 | 0.500 | 0 ==> 1 |
| *Atelerix albiventris* | 21 | 1 | 0.286 | 2 ==> 1 |
|  | 82 | 1 | 0.167 | 0 ==> 1 |
|  | 91 | 1 | 0.250 | 1 ==> 0 |
|  | 113 | 1 | 0.500 | 0 ==> 1 |
|  | 128 | 1 | 0.500 | 1 ==> 2 |
| *Atelerix algirus* | 21 | 1 | 0.286 | 2 ==> 1 |
| *Atelerix sclateri* | 82 | 1 | 0.167 | 0 ==> 1 |
| *Erinaceus amurensis* | 93 | 1 | 0.333 | 1 ==> 0 |
| *Erinaceus europaeus* | 21 | 1 | 0.286 | 2 ==> 1 |
|  | 88 | 1 | 0.333 | 0 ==> 1 |
|  | 110 | 1 | 0.333 | 0 --> 1 |
|  | 112 | 1 | 0.333 | 1 --> 0 |
| *Mesechinus dauuricus* | 130 | 1 | 1.000 | 0 --> 1 |
|  | 131 | 1 | 1.000 | 1 --> 2 |
| *Mesechinus hughi* | 32 | 1 | 0.500 | 0 ==> 1 |
|  | 92 | 1 | 0.500 | 3 ==> 4 |
|  | 93 | 1 | 0.333 | 1 ==> 0 |
|  | 97 | 1 | 1.000 | 0 ==> 2 |
|  | 98 | 1 | 0.500 | 1 ==> 2 |
|  | 100 | 1 | 0.500 | 1 ==> 0 |

**2. Strict consensus morphological tree of 23 taxon using weighted characters.**

Tree length = 428, Consistency index (CI) = 0.6612, Homoplasy index (HI) = 0.3388, CI excluding uninformative characters = 0.6437, HI excluding uninformative characters = 0.3563, Retention index (RI) = 0.8739, Rescaled consistency index (RC) = 0.5778

**
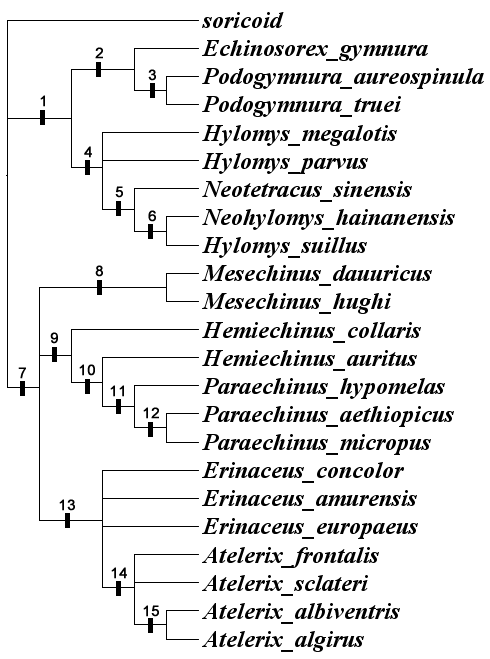
**

**Apomorphy list**

| Stem | Character | Steps | CI | Change |
| --- | --- | --- | --- | --- |
| Stem 1 | 4 | 1 | 0.500 | 0 --> 1 |
|  | 83 | 1 | 0.333 | 0 --> 1 |
|  | 91 | 1 | 0.200 | 0 --> 1 |
| Stem 2 | 8 | 1 | 0.500 | 0 --> 1 |
|  | 9 | 1 | 1.000 | 2 ==> 1 |
|  | 11 | 1 | 1.000 | 1 --> 2 |
|  | 21 | 1 | 0.333 | 0 --> 1 |
|  | 25 | 1 | 0.250 | 0 --> 1 |
|  | 41 | 1 | 0.500 | 0 --> 1 |
|  | 63 | 1 | 0.500 | 0 --> 1 |
|  | 123 | 1 | 1.000 | 0 --> 1 |
|  | 124 | 1 | 1.000 | 0 --> 1 |
|  | 125 | 1 | 1.000 | 0 --> 1 |
|  | 126 | 1 | 1.000 | 0 --> 1 |
| *Echinosorex gymnura* | 4 | 1 | 0.500 | 1 --> 0 |
|  | 5 | 1 | 0.500 | 0 --> 1 |
|  | 7 | 1 | 0.500 | 0 ==> 1 |
|  | 8 | 1 | 0.500 | 1 --> 0 |
|  | 9 | 1 | 1.000 | 1 --> 0 |
|  | 10 | 1 | 0.400 | 1 ==> 2 |
|  | 21 | 1 | 0.333 | 1 --> 0 |
|  | 25 | 1 | 0.250 | 1 --> 0 |
|  | 26 | 1 | 1.000 | 0 ==> 1 |
|  | 35 | 1 | 0.500 | 1 ==> 0 |
|  | 61 | 1 | 1.000 | 0 --> 1 |
|  | 63 | 1 | 0.500 | 1 --> 0 |
|  | 64 | 1 | 0.500 | 1 ==> 0 |
|  | 70 | 1 | 0.333 | 1 --> 0 |
|  | 82 | 1 | 0.143 | 1 ==> 0 |
|  | 83 | 1 | 0.333 | 1 --> 0 |
|  | 86 | 1 | 0.500 | 1 --> 0 |
|  | 91 | 1 | 0.200 | 1 --> 0 |
|  | 112 | 1 | 0.333 | 0 --> 1 |
|  | 121 | 1 | 1.000 | 0 --> 1 |
|  | 122 | 1 | 0.500 | 0 --> 1 |
|  | 135 | 1 | 1.000 | 0 --> 1 |
| Stem 3 | 4 | 1 | 0.500 | 1 --> 0 |
|  | 5 | 1 | 0.500 | 0 --> 1 |
|  | 9 | 1 | 1.000 | 1 --> 0 |
|  | 10 | 1 | 0.400 | 1 ==> 0 |
|  | 22 | 1 | 0.333 | 0 ==> 1 |
|  | 61 | 1 | 1.000 | 0 --> 1 |
|  | 66 | 1 | 0.500 | 0 ==> 1 |
|  | 71 | 1 | 0.250 | 0 --> 1 |
|  | 83 | 1 | 0.333 | 1 --> 0 |
|  | 135 | 1 | 1.000 | 0 --> 1 |
| *Podogymnura truei* | 42 | 1 | 0.500 | 0 ==> 1 |
|  | 59 | 1 | 0.500 | 0 ==> 1 |
| Stem 4 | 8 | 1 | 0.500 | 0 --> 1 |
|  | 9 | 1 | 1.000 | 2 --> 1 |
|  | 11 | 1 | 1.000 | 1 --> 2 |
|  | 15 | 1 | 0.500 | 1 --> 0 |
|  | 19 | 1 | 1.000 | 0 ==> 1 |
|  | 25 | 1 | 0.250 | 0 --> 1 |
|  | 41 | 1 | 0.500 | 0 --> 1 |
|  | 42 | 1 | 0.500 | 0 ==> 1 |
|  | 59 | 1 | 0.500 | 0 ==> 1 |
|  | 70 | 1 | 0.333 | 1 --> 0 |
|  | 92 | 2 | 0.500 | 2 --> 0 |
|  | 121 | 1 | 1.000 | 0 --> 1 |
|  | 123 | 1 | 1.000 | 0 --> 1 |
|  | 124 | 1 | 1.000 | 0 --> 1 |
|  | 125 | 1 | 1.000 | 0 --> 1 |
|  | 126 | 1 | 1.000 | 0 --> 1 |
| Stem 5 | 6 | 1 | 0.500 | 0 ==> 1 |
|  | 14 | 1 | 1.000 | 0 ==> 1 |
|  | 16 | 1 | 1.000 | 0 ==> 1 |
|  | 21 | 1 | 0.333 | 0 --> 1 |
|  | 71 | 1 | 0.250 | 0 --> 1 |
|  | 74 | 1 | 0.667 | 0 ==> 1 |
|  | 122 | 1 | 0.500 | 0 --> 1 |
| *Neotetracus sinensis* | 1 | 1 | 0.333 | 0 ==> 1 |
|  | 70 | 1 | 0.333 | 0 ==> 1 |
|  | 98 | 1 | 0.500 | 1 ==> 2 |
|  | 100 | 1 | 0.500 | 1 ==> 0 |
| Stem 6 | 25 | 1 | 0.250 | 1 ==> 0 |
|  | 86 | 1 | 0.500 | 1 --> 0 |
|  | 88 | 1 | 0.333 | 0 --> 1 |
|  | 91 | 1 | 0.200 | 1 --> 0 |
|  | 109 | 1 | 0.500 | 0 --> 1 |
| *Neohylomys hainanensis* | 63 | 1 | 0.500 | 0 ==> 1 |
| *Hylomys suillus* | 71 | 1 | 0.250 | 1 --> 0 |
| *Hylomys megalotis* | 1 | 1 | 0.333 | 0 ==> 1 |
|  | 4 | 1 | 0.500 | 1 --> 0 |
|  | 5 | 1 | 0.500 | 0 ==> 1 |
|  | 10 | 1 | 0.400 | 1 ==> 2 |
|  | 17 | 1 | 0.500 | 0 ==> 1 |
|  | 82 | 1 | 0.143 | 1 ==> 0 |
|  | 83 | 1 | 0.333 | 1 --> 0 |
| *Hylomys parvus* | 15 | 1 | 0.500 | 0 --> 1 |
| Stem 7 | 1 | 1 | 0.333 | 0 ==> 1 |
|  | 2 | 1 | 1.000 | 0 ==> 1 |
|  | 4 | 2 | 0.500 | 0 ==> 2 |
|  | 6 | 1 | 0.500 | 0 ==> 1 |
|  | 7 | 1 | 0.500 | 0 ==> 1 |
|  | 10 | 1 | 0.400 | 1 ==> 2 |
|  | 17 | 1 | 0.500 | 0 ==> 1 |
|  | 18 | 1 | 1.000 | 0 ==> 1 |
|  | 21 | 1 | 0.333 | 0 --> 2 |
|  | 22 | 1 | 0.333 | 0 ==> 1 |
|  | 23 | 1 | 0.250 | 0 --> 1 |
|  | 24 | 1 | 1.000 | 0 ==> 1 |
|  | 27 | 1 | 1.000 | 0 ==> 1 |
|  | 28 | 1 | 1.000 | 0 ==> 1 |
|  | 30 | 1 | 1.000 | 0 ==> 1 |
|  | 33 | 1 | 1.000 | 0 ==> 1 |
|  | 36 | 1 | 1.000 | 0 --> 2 |
|  | 40 | 1 | 1.000 | 0 ==> 1 |
|  | 46 | 1 | 1.000 | 0 ==> 1 |
|  | 47 | 1 | 1.000 | 0 ==> 1 |
|  | 58 | 1 | 1.000 | 0 ==> 1 |
|  | 60 | 1 | 1.000 | 0 ==> 1 |
|  | 63 | 1 | 0.500 | 0 --> 2 |
|  | 64 | 1 | 0.500 | 1 ==> 0 |
|  | 66 | 1 | 0.500 | 0 ==> 1 |
|  | 71 | 1 | 0.250 | 0 --> 1 |
|  | 74 | 2 | 0.667 | 0 ==> 2 |
|  | 82 | 1 | 0.143 | 1 ==> 0 |
|  | 83 | 1 | 0.333 | 0 --> 1 |
|  | 91 | 1 | 0.200 | 0 --> 1 |
|  | 92 | 1 | 0.500 | 2 --> 3 |
|  | 95 | 1 | 1.000 | 0 ==> 1 |
|  | 114 | 1 | 1.000 | 0 ==> 1 |
|  | 128 | 1 | 0.500 | 0 --> 1 |
|  | 129 | 1 | 1.000 | 0 ==> 1 |
|  | 131 | 1 | 1.000 | 0 --> 1 |
|  | 132 | 1 | 1.000 | 0 ==> 1 |
|  | 133 | 1 | 1.000 | 0 ==> 3 |
|  | 134 | 1 | 0.500 | 0 --> 1 |
| Stem 9 | 3 | 1 | 1.000 | 0 ==> 1 |
|  | 30 | 1 | 1.000 | 1 ==> 2 |
|  | 34 | 1 | 1.000 | 0 ==> 1 |
|  | 127 | 1 | 1.000 | 0 ==> 1 |
|  | 130 | 1 | 1.000 | 0 --> 2 |
|  | 131 | 1 | 1.000 | 1 --> 3 |
|  | 134 | 1 | 0.500 | 1 --> 0 |
| Stem 10 | 23 | 1 | 0.250 | 1 --> 0 |
| Stem 11 | 12 | 1 | 1.000 | 1 ==> 2 |
|  | 30 | 1 | 1.000 | 2 ==> 3 |
|  | 43 | 1 | 1.000 | 0 ==> 1 |
|  | 44 | 1 | 1.000 | 0 ==> 1 |
|  | 133 | 1 | 1.000 | 3 ==> 2 |
| Stem 12 | 30 | 1 | 1.000 | 3 ==> 4 |
|  | 82 | 1 | 0.143 | 0 ==> 1 |
|  | 91 | 1 | 0.200 | 1 --> 0 |
|  | 92 | 3 | 0.500 | 3 --> 0 |
|  | 100 | 1 | 0.500 | 1 --> 0 |
|  | 109 | 1 | 0.500 | 0 --> 1 |
| *Paraechinus aethiopicus* | 12 | 1 | 1.000 | 2 ==> 3 |
| *Paraechinus micropus* | 22 | 1 | 0.333 | 1 ==> 0 |
|  | 23 | 1 | 0.250 | 0 ==> 1 |
| *Paraechinus hypomelas* | 88 | 1 | 0.333 | 0 ==> 1 |
| *Hemiechinus auritus* | 93 | 1 | 0.333 | 1 ==> 0 |
| Stem 8 | 11 | 1 | 1.000 | 1 ==> 0 |
|  | 13 | 1 | 0.500 | 1 ==> 0 |
|  | 31 | 1 | 1.000 | 0 ==> 1 |
|  | 41 | 1 | 0.500 | 0 ==> 1 |
|  | 91 | 1 | 0.200 | 1 --> 0 |
|  | 92 | 1 | 0.500 | 3 --> 4 |
|  | 93 | 1 | 0.333 | 1 --> 0 |
|  | 97 | 1 | 1.000 | 0 --> 2 |
|  | 98 | 1 | 0.500 | 1 --> 2 |
|  | 100 | 1 | 0.500 | 1 --> 0 |
|  | 130 | 1 | 1.000 | 0 --> 1 |
|  | 131 | 1 | 1.000 | 1 --> 2 |
| *Mesechinus hughi* | 23 | 1 | 0.250 | 1 --> 0 |
|  | 32 | 1 | 0.500 | 0 ==> 1 |
|  | 82 | 1 | 0.143 | 0 ==> 1 |
| Stem 13 | 32 | 1 | 0.500 | 0 ==> 1 |
|  | 110 | 1 | 0.333 | 1 --> 0 |
|  | 112 | 1 | 0.333 | 0 --> 1 |
|  | 128 | 1 | 0.500 | 1 --> 0 |
|  | 133 | 1 | 1.000 | 3 ==> 1 |
| Stem 14 | 25 | 1 | 0.250 | 0 ==> 1 |
|  | 29 | 1 | 1.000 | 0 ==> 1 |
|  | 73 | 1 | 1.000 | 1 ==> 0 |
|  | 92 | 1 | 0.500 | 3 --> 2 |
|  | 128 | 1 | 0.500 | 0 ==> 1 |
| Stem 15 | 21 | 1 | 0.333 | 2 ==> 1 |
| *Atelerix albiventris* | 82 | 1 | 0.143 | 0 ==> 1 |
|  | 91 | 1 | 0.200 | 1 ==> 0 |
|  | 113 | 1 | 0.500 | 0 ==> 1 |
|  | 128 | 1 | 0.500 | 1 ==> 2 |
| *Atelerix scalteri* | 82 | 1 | 0.143 | 0 ==> 1 |
| *Erinaceus amurensis* | 93 | 1 | 0.333 | 1 ==> 0 |
| *Erinaceus europaeus* | 21 | 1 | 0.333 | 2 ==> 1 |
|  | 88 | 1 | 0.333 | 0 ==> 1 |
|  | 110 | 1 | 0.333 | 0 --> 1 |
|  | 112 | 1 | 0.333 | 1 --> 0 |

**3. Strict consensus morphological tree of 23 taxon based on non-dental characters.**

Tree length = 122, Consistency index (CI) = 0.6803, Homoplasy index (HI) = 0.3197, CI excluding uninformative characters = 0.6667, HI excluding uninformative characters = 0.3333, Retention index (RI) = 0.8892, Rescaled consistency index (RC) = 0.6050

**
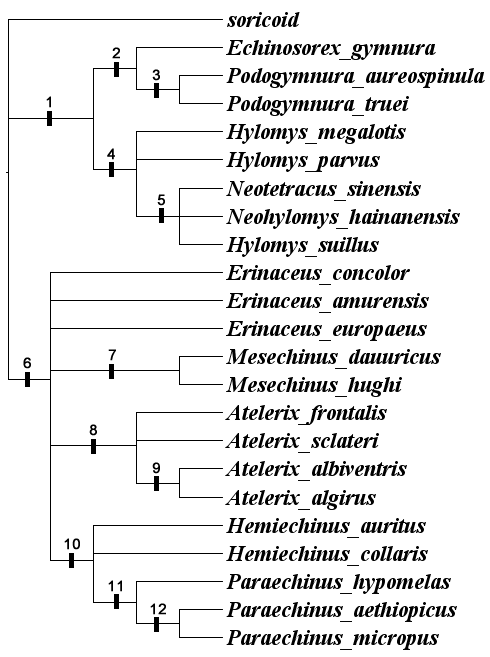
**

Apomorphy list

| Branch | Character | Steps | CI | Change |
| --- | --- | --- | --- | --- |
| Stem 1 | 4 | 1 | 0.500 | 0 --> 1 |
| Stem 2 | 8 | 1 | 0.500 | 0 --> 1 |
|  | 9 | 1 | 1.000 | 2 ==> 1 |
|  | 11 | 1 | 1.000 | 1 --> 2 |
|  | 21 | 1 | 0.333 | 0 --> 1 |
|  | 25 | 1 | 0.200 | 0 --> 1 |
|  | 41 | 1 | 0.500 | 0 --> 1 |
|  | 123 | 1 | 1.000 | 0 --> 1 |
|  | 124 | 1 | 1.000 | 0 --> 1 |
|  | 125 | 1 | 1.000 | 0 --> 1 |
|  | 126 | 1 | 1.000 | 0 --> 1 |
| *Echinosorex gymnura* | 4 | 1 | 0.500 | 1 --> 0 |
|  | 5 | 1 | 0.500 | 0 --> 1 |
|  | 7 | 1 | 0.500 | 0 ==> 1 |
|  | 8 | 1 | 0.500 | 1 --> 0 |
|  | 9 | 1 | 1.000 | 1 --> 0 |
|  | 10 | 1 | 0.400 | 1 ==> 2 |
|  | 21 | 1 | 0.333 | 1 --> 0 |
|  | 25 | 1 | 0.200 | 1 --> 0 |
|  | 26 | 1 | 1.000 | 0 ==> 1 |
|  | 35 | 1 | 0.500 | 1 ==> 0 |
|  | 61 | 1 | 1.000 | 0 --> 1 |
|  | 121 | 1 | 1.000 | 0 --> 1 |
|  | 122 | 1 | 0.500 | 0 --> 1 |
|  | 135 | 1 | 1.000 | 0 --> 1 |
| Stem 3 | 4 | 1 | 0.500 | 1 --> 0 |
|  | 5 | 1 | 0.500 | 0 --> 1 |
|  | 9 | 1 | 1.000 | 1 --> 0 |
|  | 10 | 1 | 0.400 | 1 ==> 0 |
|  | 22 | 1 | 0.333 | 0 ==> 1 |
|  | 61 | 1 | 1.000 | 0 --> 1 |
|  | 135 | 1 | 1.000 | 0 --> 1 |
| *Podogymnura truei* | 42 | 1 | 0.500 | 0 ==> 1 |
|  | 59 | 1 | 0.500 | 0 ==> 1 |
| Stem 4 | 8 | 1 | 0.500 | 0 --> 1 |
|  | 9 | 1 | 1.000 | 2 --> 1 |
|  | 11 | 1 | 1.000 | 1 --> 2 |
|  | 15 | 1 | 0.500 | 1 --> 0 |
|  | 19 | 1 | 1.000 | 0 ==> 1 |
|  | 25 | 1 | 0.200 | 0 --> 1 |
|  | 41 | 1 | 0.500 | 0 --> 1 |
|  | 42 | 1 | 0.500 | 0 ==> 1 |
|  | 59 | 1 | 0.500 | 0 ==> 1 |
|  | 121 | 1 | 1.000 | 0 --> 1 |
|  | 123 | 1 | 1.000 | 0 --> 1 |
|  | 124 | 1 | 1.000 | 0 --> 1 |
|  | 125 | 1 | 1.000 | 0 --> 1 |
|  | 126 | 1 | 1.000 | 0 --> 1 |
| Stem 5 | 6 | 1 | 0.500 | 0 ==> 1 |
|  | 14 | 1 | 1.000 | 0 ==> 1 |
|  | 16 | 1 | 1.000 | 0 ==> 1 |
|  | 21 | 1 | 0.333 | 0 --> 1 |
|  | 25 | 1 | 0.200 | 1 --> 0 |
|  | 122 | 1 | 0.500 | 0 --> 1 |
| *Neotetracus sinensis* | 1 | 1 | 0.333 | 0 ==> 1 |
|  | 25 | 1 | 0.200 | 0 --> 1 |
| *Hylomys megalotis* | 1 | 1 | 0.333 | 0 ==> 1 |
|  | 4 | 1 | 0.500 | 1 --> 0 |
|  | 5 | 1 | 0.500 | 0 ==> 1 |
|  | 10 | 1 | 0.400 | 1 ==> 2 |
|  | 17 | 1 | 0.500 | 0 ==> 1 |
| *Hylomys parvus* | 15 | 1 | 0.500 | 0 --> 1 |
| Stem 6 | 1 | 1 | 0.333 | 0 ==> 1 |
|  | 2 | 1 | 1.000 | 0 ==> 1 |
|  | 4 | 2 | 0.500 | 0 ==> 2 |
|  | 6 | 1 | 0.500 | 0 ==> 1 |
|  | 7 | 1 | 0.500 | 0 ==> 1 |
|  | 10 | 1 | 0.400 | 1 ==> 2 |
|  | 17 | 1 | 0.500 | 0 ==> 1 |
|  | 18 | 1 | 1.000 | 0 ==> 1 |
|  | 21 | 1 | 0.333 | 0 --> 2 |
|  | 22 | 1 | 0.333 | 0 ==> 1 |
|  | 23 | 1 | 0.200 | 0 ==> 1 |
|  | 24 | 1 | 1.000 | 0 ==> 1 |
|  | 27 | 1 | 1.000 | 0 ==> 1 |
|  | 28 | 1 | 1.000 | 0 ==> 1 |
|  | 30 | 1 | 1.000 | 0 ==> 1 |
|  | 32 | 1 | 0.333 | 0 ==> 1 |
|  | 33 | 1 | 1.000 | 0 ==> 1 |
|  | 36 | 1 | 1.000 | 0 --> 2 |
|  | 40 | 1 | 1.000 | 0 ==> 1 |
|  | 46 | 1 | 1.000 | 0 ==> 1 |
|  | 47 | 1 | 1.000 | 0 ==> 1 |
|  | 58 | 1 | 1.000 | 0 ==> 1 |
|  | 60 | 1 | 1.000 | 0 ==> 1 |
|  | 129 | 1 | 1.000 | 0 ==> 1 |
|  | 131 | 1 | 1.000 | 0 ==> 1 |
|  | 132 | 1 | 1.000 | 0 ==> 1 |
|  | 133 | 1 | 0.750 | 0 ==> 1 |
|  | 134 | 1 | 0.500 | 0 ==> 1 |
| Stem 10 | 3 | 1 | 1.000 | 0 ==> 1 |
|  | 23 | 1 | 0.200 | 1 --> 0 |
|  | 30 | 1 | 1.000 | 1 ==> 2 |
|  | 32 | 1 | 0.333 | 1 ==> 0 |
|  | 34 | 1 | 1.000 | 0 ==> 1 |
|  | 127 | 1 | 1.000 | 0 ==> 1 |
|  | 128 | 1 | 0.500 | 0 ==> 1 |
|  | 130 | 1 | 1.000 | 0 ==> 2 |
|  | 131 | 1 | 1.000 | 1 ==> 3 |
|  | 133 | 1 | 0.750 | 1 ==> 3 |
|  | 134 | 1 | 0.500 | 1 ==> 0 |
| Stem 11 | 12 | 1 | 1.000 | 1 ==> 2 |
|  | 30 | 1 | 1.000 | 2 ==> 3 |
|  | 43 | 1 | 1.000 | 0 ==> 1 |
|  | 44 | 1 | 1.000 | 0 ==> 1 |
|  | 133 | 1 | 0.750 | 3 ==> 2 |
| Stem 12 | 30 | 1 | 1.000 | 3 ==> 4 |
| *Paraechinus aethiopicus* | 12 | 1 | 1.000 | 2 ==> 3 |
| *Paraechinus micropus* | 22 | 1 | 0.333 | 1 ==> 0 |
|  | 23 | 1 | 0.200 | 0 --> 1 |
| *Hemiechinus collaris* | 23 | 1 | 0.200 | 0 --> 1 |
| Stem 7 | 11 | 1 | 1.000 | 1 ==> 0 |
|  | 13 | 1 | 0.500 | 1 ==> 0 |
|  | 31 | 1 | 1.000 | 0 ==> 1 |
|  | 41 | 1 | 0.500 | 0 ==> 1 |
|  | 128 | 1 | 0.500 | 0 --> 1 |
|  | 130 | 1 | 1.000 | 0 --> 1 |
|  | 131 | 1 | 1.000 | 1 --> 2 |
|  | 133 | 1 | 0.750 | 1 --> 3 |
| *Mesechinus dauuricus* | 32 | 1 | 0.333 | 1 ==> 0 |
| *Mesechinus hughi* | 23 | 1 | 0.200 | 1 ==> 0 |
| Stem 8 | 25 | 1 | 0.200 | 0 ==> 1 |
|  | 29 | 1 | 1.000 | 0 ==> 1 |
|  | 128 | 1 | 0.500 | 0 ==> 1 |
| Stem 9 | 21 | 1 | 0.333 | 2 ==> 1 |
| *Atelerix albiventris* | 128 | 1 | 0.500 | 1 ==> 2 |
| *Erinaceus europaeus* | 21 | 1 | 0.333 | 2 ==> 1 |

**4. Strict consensus morphological tree of 23 taxon based on only dental characters.**

Tree length = 60, Consistency index (CI) = 0.5667, Homoplasy index (HI) = 0.4333, CI excluding uninformative characters = 0.5185, HI excluding uninformative characters = 0.4815, Retention index (RI) = 0.7234, Rescaled consistency index (RC) = 0.4099


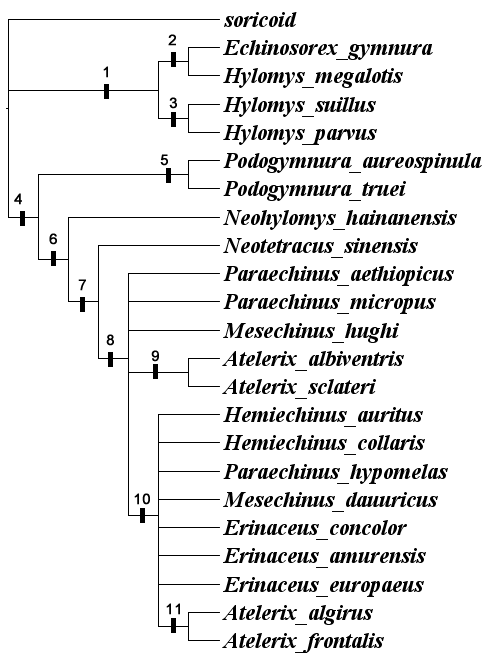


**Apomorphy list**

| Branch | Character | Steps | CI | Change |
| --- | --- | --- | --- | --- |
| Stem 2 | 63 | 1 | 1.000 | 1 --> 0 |
|  | 70 | 1 | 0.500 | 1 --> 0 |
| *Echinosorex gymnura* | 64 | 1 | 0.500 | 1 --> 0 |
|  | 82 | 1 | 0.500 | 1 --> 0 |
|  | 86 | 1 | 1.000 | 1 --> 0 |
|  | 92 | 2 | 0.400 | 0 --> 2 |
|  | 100 | 1 | 0.500 | 0 --> 1 |
|  | 112 | 1 | 0.333 | 0 --> 1 |
| *Hylomys megalotis* | 82 | 1 | 0.500 | 1 --> 0 |
| Stem 3 | 63 | 1 | 1.000 | 1 --> 0 |
|  | 70 | 1 | 0.500 | 1 --> 0 |
|  | 83 | 1 | 0.500 | 0 ==> 1 |
|  | 86 | 1 | 1.000 | 1 --> 0 |
|  | 88 | 1 | 0.333 | 0 --> 1 |
|  | 100 | 1 | 0.500 | 0 --> 1 |
|  | 109 | 1 | 0.500 | 0 --> 1 |
| Stem 4 | 71 | 1 | 1.000 | 0 ==> 1 |
| Stem 5 | 66 | 1 | 0.500 | 0 ==> 1 |
| Stem 6 | 74 | 1 | 1.000 | 0 ==> 1 |
|  | 83 | 1 | 0.500 | 0 ==> 1 |
| Stem 7 | 63 | 1 | 1.000 | 1 ==> 2 |
| *Neotetracus sinensis* | 91 | 1 | 0.500 | 0 ==> 1 |
|  | 98 | 1 | 0.500 | 1 ==> 2 |
| Stem 8 | 64 | 1 | 0.500 | 1 ==> 0 |
|  | 66 | 1 | 0.500 | 0 ==> 1 |
|  | 74 | 1 | 1.000 | 1 ==> 2 |
|  | 92 | 3 | 0.400 | 0 --> 3 |
|  | 95 | 1 | 1.000 | 0 ==> 1 |
|  | 114 | 1 | 1.000 | 0 ==> 1 |
| *Paraechinus aethiopicus* | 92 | 3 | 0.400 | 3 --> 0 |
|  | 109 | 1 | 0.500 | 0 ==> 1 |
| Stem 10 | 82 | 1 | 0.500 | 1 ==> 0 |
|  | 91 | 1 | 0.500 | 0 ==> 1 |
|  | 100 | 1 | 0.500 | 0 --> 1 |
| *Hemiechinus auritus* | 93 | 1 | 0.333 | 1 ==> 0 |
| *Paraechinus hypomelas* | 88 | 1 | 0.333 | 0 ==> 1 |
| Stem 11 | 73 | 1 | 0.500 | 1 ==> 0 |
|  | 92 | 1 | 0.400 | 3 --> 2 |
|  | 110 | 1 | 0.333 | 1 --> 0 |
|  | 112 | 1 | 0.333 | 0 --> 1 |
| *Erinaceus amurensis* | 93 | 1 | 0.333 | 1 ==> 0 |
|  | 110 | 1 | 0.333 | 1 ==> 0 |
|  | 112 | 1 | 0.333 | 0 ==> 1 |
| *Erinaceus europaeus* | 88 | 1 | 0.333 | 0 ==> 1 |
| Stem 9 | 73 | 1 | 0.500 | 1 ==> 0 |
|  | 100 | 1 | 0.500 | 0 --> 1 |
|  | 113 | 1 | 0.500 | 0 --> 1 |
| *Mesechinus hughi* | 92 | 1 | 0.400 | 3 ==> 4 |
|  | 93 | 1 | 0.333 | 1 ==> 0 |
|  | 97 | 1 | 1.000 | 0 ==> 2 |
|  | 98 | 1 | 0.500 | 1 ==> 2 |
| *Neohylomys hainanensis* | 70 | 1 | 0.500 | 1 ==> 0 |

**5. Strict consensus morphological tree of 15 taxon using unweighted characters.**

Tree length = 173, Consistency index (CI) = 0.6763, Homoplasy index (HI) = 0.3237, CI excluding uninformative characters = 0.6387, HI excluding uninformative characters = 0.3613, Retention index (RI) = 0.8069, Rescaled consistency index (RC) = 0.5457


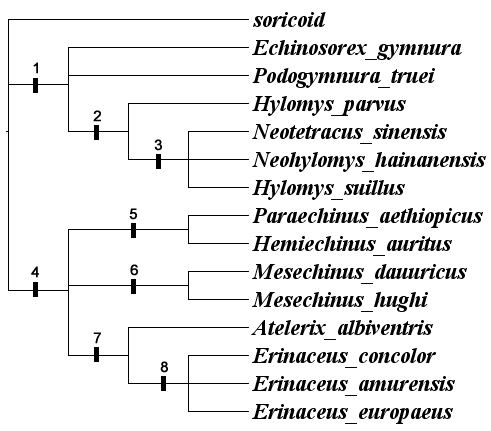


**Apomorphy list**

| Branch | Character | Steps | CI | Change |
| --- | --- | --- | --- | --- |
| Stem 1 | 8 | 1 | 0.500 | 0 --> 1 |
|  | 9 | 1 | 0.750 | 2 ==> 1 |
|  | 11 | 1 | 1.000 | 1 --> 2 |
|  | 41 | 1 | 0.500 | 0 ==> 1 |
|  | 42 | 1 | 0.500 | 0 --> 1 |
|  | 59 | 1 | 0.500 | 0 --> 1 |
|  | 63 | 1 | 0.500 | 0 --> 1 |
|  | 123 | 1 | 1.000 | 0 ==> 1 |
|  | 124 | 1 | 1.000 | 0 ==> 1 |
|  | 125 | 1 | 1.000 | 0 ==> 1 |
|  | 126 | 1 | 1.000 | 0 ==> 1 |
| *Echinosorex gymnura* | 5 | 1 | 0.500 | 0 --> 1 |
|  | 7 | 1 | 0.500 | 0 ==> 1 |
|  | 8 | 1 | 0.500 | 1 --> 0 |
|  | 9 | 1 | 0.750 | 1 --> 0 |
|  | 10 | 1 | 0.500 | 1 ==> 2 |
|  | 21 | 1 | 0.400 | 1 ==> 0 |
|  | 26 | 1 | 1.000 | 0 ==> 1 |
|  | 35 | 1 | 0.500 | 1 ==> 0 |
|  | 42 | 1 | 0.500 | 1 --> 0 |
|  | 59 | 1 | 0.500 | 1 --> 0 |
|  | 61 | 1 | 0.500 | 0 --> 1 |
|  | 63 | 1 | 0.500 | 1 --> 0 |
|  | 64 | 1 | 0.500 | 1 ==> 0 |
|  | 70 | 1 | 0.333 | 1 --> 0 |
|  | 82 | 1 | 0.250 | 1 ==> 0 |
|  | 86 | 1 | 0.500 | 1 --> 0 |
|  | 112 | 1 | 0.500 | 0 ==> 1 |
|  | 121 | 1 | 1.000 | 0 --> 1 |
|  | 122 | 1 | 1.000 | 0 --> 1 |
|  | 135 | 1 | 0.500 | 0 --> 1 |
| *Podogymnura truei* | 5 | 1 | 0.500 | 0 --> 1 |
|  | 9 | 1 | 0.750 | 1 --> 0 |
|  | 10 | 1 | 0.500 | 1 ==> 0 |
|  | 22 | 1 | 0.500 | 0 ==> 1 |
|  | 25 | 1 | 0.250 | 0 ==> 1 |
|  | 61 | 1 | 0.500 | 0 --> 1 |
|  | 66 | 1 | 0.500 | 0 ==> 1 |
|  | 135 | 1 | 0.500 | 0 --> 1 |
| Stem 2 | 4 | 1 | 0.667 | 0 ==> 1 |
|  | 19 | 1 | 1.000 | 0 ==> 1 |
|  | 63 | 1 | 0.500 | 1 --> 0 |
|  | 70 | 1 | 0.333 | 1 --> 0 |
|  | 83 | 1 | 0.500 | 0 ==> 1 |
|  | 92 | 2 | 0.571 | 2 --> 0 |
|  | 121 | 1 | 1.000 | 0 --> 1 |
|  | 122 | 1 | 1.000 | 0 --> 1 |
| Stem 3 | 6 | 1 | 0.500 | 0 ==> 1 |
|  | 14 | 1 | 1.000 | 0 ==> 1 |
|  | 15 | 1 | 1.000 | 1 ==> 0 |
|  | 16 | 1 | 1.000 | 0 ==> 1 |
|  | 74 | 1 | 0.667 | 0 ==> 1 |
| *Neotetracus sinensis* | 1 | 1 | 0.500 | 0 ==> 1 |
|  | 25 | 1 | 0.250 | 0 ==> 1 |
|  | 70 | 1 | 0.333 | 0 --> 1 |
|  | 91 | 1 | 0.333 | 0 ==> 1 |
|  | 98 | 1 | 0.500 | 1 ==> 2 |
|  | 100 | 1 | 0.500 | 1 ==> 0 |
| *Neohylomys hainanensis* | 63 | 1 | 0.500 | 0 ==> 1 |
| *Hylomys suillus* | 71 | 1 | 0.333 | 1 ==> 0 |
|  | 86 | 1 | 0.500 | 1 --> 0 |
|  | 88 | 1 | 0.500 | 0 ==> 1 |
|  | 109 | 1 | 0.500 | 0 ==> 1 |
| *Hylomys parvus* | 21 | 1 | 0.400 | 1 ==> 0 |
|  | 25 | 1 | 0.250 | 0 ==> 1 |
|  | 71 | 1 | 0.333 | 1 ==> 0 |
| Stem 4 | 1 | 1 | 0.500 | 0 ==> 1 |
|  | 2 | 1 | 1.000 | 0 ==> 1 |
|  | 4 | 2 | 0.667 | 0 ==> 2 |
|  | 6 | 1 | 0.500 | 0 ==> 1 |
|  | 7 | 1 | 0.500 | 0 ==> 1 |
|  | 10 | 1 | 0.500 | 1 ==> 2 |
|  | 17 | 1 | 1.000 | 0 ==> 1 |
|  | 18 | 1 | 1.000 | 0 ==> 1 |
|  | 21 | 1 | 0.400 | 1 ==> 2 |
|  | 22 | 1 | 0.500 | 0 ==> 1 |
|  | 24 | 1 | 1.000 | 0 ==> 1 |
|  | 27 | 1 | 1.000 | 0 ==> 1 |
|  | 28 | 1 | 1.000 | 0 ==> 1 |
|  | 30 | 1 | 1.000 | 0 ==> 1 |
|  | 33 | 1 | 1.000 | 0 ==> 1 |
|  | 36 | 1 | 1.000 | 0 --> 2 |
|  | 40 | 1 | 1.000 | 0 ==> 1 |
|  | 46 | 1 | 1.000 | 0 ==> 1 |
|  | 47 | 1 | 1.000 | 0 ==> 1 |
|  | 58 | 1 | 1.000 | 0 ==> 1 |
|  | 60 | 1 | 1.000 | 0 ==> 1 |
|  | 63 | 1 | 0.500 | 0 --> 2 |
|  | 64 | 1 | 0.500 | 1 ==> 0 |
|  | 66 | 1 | 0.500 | 0 ==> 1 |
|  | 74 | 2 | 0.667 | 0 ==> 2 |
|  | 83 | 1 | 0.500 | 0 ==> 1 |
|  | 92 | 1 | 0.571 | 2 --> 3 |
|  | 95 | 1 | 1.000 | 0 ==> 1 |
|  | 114 | 1 | 1.000 | 0 ==> 1 |
|  | 128 | 1 | 0.667 | 0 ==> 1 |
|  | 129 | 1 | 1.000 | 0 ==> 1 |
|  | 131 | 1 | 1.000 | 0 --> 1 |
|  | 132 | 1 | 1.000 | 0 ==> 1 |
|  | 133 | 1 | 1.000 | 0 ==> 3 |
|  | 134 | 1 | 0.500 | 0 --> 1 |
| Stem 5 | 3 | 1 | 1.000 | 0 ==> 1 |
|  | 30 | 1 | 1.000 | 1 ==> 2 |
|  | 34 | 1 | 1.000 | 0 ==> 1 |
|  | 127 | 1 | 1.000 | 0 ==> 1 |
|  | 130 | 1 | 1.000 | 0 --> 2 |
|  | 131 | 1 | 1.000 | 1 --> 3 |
|  | 134 | 1 | 0.500 | 1 --> 0 |
| *Paraechinus aethiopicus* | 12 | 2 | 1.000 | 1 ==> 3 |
|  | 30 | 2 | 1.000 | 2 ==> 4 |
|  | 43 | 1 | 1.000 | 0 ==> 1 |
|  | 44 | 1 | 1.000 | 0 ==> 1 |
|  | 92 | 3 | 0.571 | 3 ==> 0 |
|  | 100 | 1 | 0.500 | 1 ==> 0 |
|  | 109 | 1 | 0.500 | 0 ==> 1 |
|  | 133 | 1 | 1.000 | 3 ==> 2 |
| *Hemiechinus auritus* | 82 | 1 | 0.250 | 1 ==> 0 |
|  | 91 | 1 | 0.333 | 0 ==> 1 |
|  | 93 | 1 | 0.333 | 1 ==> 0 |
| Stem 6 | 11 | 1 | 1.000 | 1 ==> 0 |
|  | 13 | 1 | 0.500 | 1 ==> 0 |
|  | 31 | 1 | 1.000 | 0 ==> 1 |
|  | 41 | 1 | 0.500 | 0 ==> 1 |
|  | 92 | 1 | 0.571 | 3 --> 4 |
|  | 93 | 1 | 0.333 | 1 --> 0 |
|  | 97 | 1 | 1.000 | 0 --> 2 |
|  | 98 | 1 | 0.500 | 1 --> 2 |
|  | 100 | 1 | 0.500 | 1 --> 0 |
|  | 130 | 1 | 1.000 | 0 --> 1 |
|  | 131 | 1 | 1.000 | 1 --> 2 |
| *Mesechinus dauuricus* | 23 | 1 | 0.500 | 0 ==> 1 |
|  | 82 | 1 | 0.250 | 1 ==> 0 |
| *Mesechinus hughi* | 32 | 1 | 0.500 | 0 ==> 1 |
| Stem 7 | 23 | 1 | 0.500 | 0 ==> 1 |
|  | 32 | 1 | 0.500 | 0 ==> 1 |
|  | 133 | 1 | 1.000 | 3 ==> 1 |
| *Atelerix albiventris* | 21 | 1 | 0.400 | 2 ==> 1 |
|  | 25 | 1 | 0.250 | 0 ==> 1 |
|  | 29 | 1 | 1.000 | 0 ==> 1 |
|  | 73 | 1 | 1.000 | 1 ==> 0 |
|  | 113 | 1 | 0.500 | 0 ==> 1 |
|  | 128 | 1 | 0.667 | 1 ==> 2 |
| Stem 8 | 82 | 1 | 0.250 | 1 ==> 0 |
|  | 91 | 1 | 0.333 | 0 ==> 1 |
|  | 128 | 1 | 0.667 | 1 ==> 0 |
| *Erinaceus amurensis* | 93 | 1 | 0.333 | 1 ==> 0 |
|  | 110 | 1 | 0.500 | 1 ==> 0 |
|  | 112 | 1 | 0.500 | 0 ==> 1 |
| *Erinaceus europaeus* | 21 | 1 | 0.400 | 2 ==> 1 |
|  | 88 | 1 | 0.500 | 0 ==> 1 |

**6. Strict consensus morphological tree of 15 taxon using weighted characters.**

Tree length = 393, Consistency index (CI) = 0.7201, Homoplasy index (HI) = 0.2799, CI excluding uninformative characters = 0.6884, HI excluding uninformative characters = 0.3116, Retention index (RI) = 0.8514, Rescaled consistency index (RC) = 0.6131


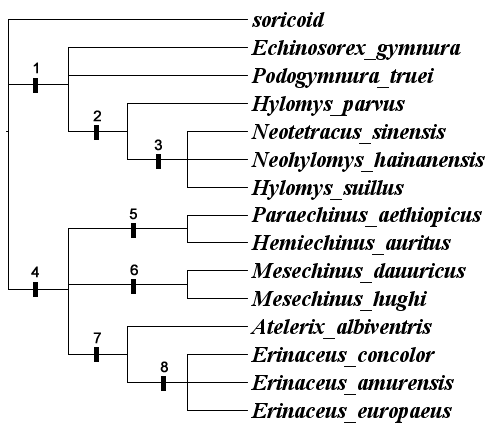


**Apomorphy list**

| Branch | Character | Steps | CI | Change |
| --- | --- | --- | --- | --- |
| Stem 1 | 8 | 1 | 0.500 | 0 --> 1 |
|  | 9 | 1 | 0.750 | 2 ==> 1 |
|  | 11 | 1 | 1.000 | 1 --> 2 |
|  | 41 | 1 | 0.500 | 0 ==> 1 |
|  | 42 | 1 | 0.500 | 0 --> 1 |
|  | 59 | 1 | 0.500 | 0 --> 1 |
|  | 63 | 1 | 0.500 | 0 --> 1 |
|  | 123 | 1 | 1.000 | 0 ==> 1 |
|  | 124 | 1 | 1.000 | 0 ==> 1 |
|  | 125 | 1 | 1.000 | 0 ==> 1 |
|  | 126 | 1 | 1.000 | 0 ==> 1 |
| *Echinosorex gymnura* | 5 | 1 | 0.500 | 0 --> 1 |
|  | 7 | 1 | 0.500 | 0 ==> 1 |
|  | 8 | 1 | 0.500 | 1 --> 0 |
|  | 9 | 1 | 0.750 | 1 --> 0 |
|  | 10 | 1 | 0.500 | 1 ==> 2 |
|  | 21 | 1 | 0.400 | 1 ==> 0 |
|  | 26 | 1 | 1.000 | 0 ==> 1 |
|  | 35 | 1 | 0.500 | 1 ==> 0 |
|  | 42 | 1 | 0.500 | 1 --> 0 |
|  | 59 | 1 | 0.500 | 1 --> 0 |
|  | 61 | 1 | 0.500 | 0 --> 1 |
|  | 63 | 1 | 0.500 | 1 --> 0 |
|  | 64 | 1 | 0.500 | 1 ==> 0 |
|  | 70 | 1 | 0.333 | 1 --> 0 |
|  | 82 | 1 | 0.250 | 1 ==> 0 |
|  | 86 | 1 | 0.500 | 1 --> 0 |
|  | 112 | 1 | 0.500 | 0 ==> 1 |
|  | 121 | 1 | 1.000 | 0 --> 1 |
|  | 122 | 1 | 1.000 | 0 --> 1 |
|  | 135 | 1 | 0.500 | 0 --> 1 |
| *Podogymnura truei* | 5 | 1 | 0.500 | 0 --> 1 |
|  | 9 | 1 | 0.750 | 1 --> 0 |
|  | 10 | 1 | 0.500 | 1 ==> 0 |
|  | 22 | 1 | 0.500 | 0 ==> 1 |
|  | 25 | 1 | 0.250 | 0 ==> 1 |
|  | 61 | 1 | 0.500 | 0 --> 1 |
|  | 66 | 1 | 0.500 | 0 ==> 1 |
|  | 135 | 1 | 0.500 | 0 --> 1 |
| Stem 2 | 4 | 1 | 0.667 | 0 ==> 1 |
|  | 19 | 1 | 1.000 | 0 ==> 1 |
|  | 63 | 1 | 0.500 | 1 --> 0 |
|  | 70 | 1 | 0.333 | 1 --> 0 |
|  | 83 | 1 | 0.500 | 0 ==> 1 |
|  | 92 | 2 | 0.571 | 2 --> 0 |
|  | 121 | 1 | 1.000 | 0 --> 1 |
|  | 122 | 1 | 1.000 | 0 --> 1 |
| Stem 3 | 6 | 1 | 0.500 | 0 ==> 1 |
|  | 14 | 1 | 1.000 | 0 ==> 1 |
|  | 15 | 1 | 1.000 | 1 ==> 0 |
|  | 16 | 1 | 1.000 | 0 ==> 1 |
|  | 74 | 1 | 0.667 | 0 ==> 1 |
| *Neotetracus sinensis* | 1 | 1 | 0.500 | 0 ==> 1 |
|  | 25 | 1 | 0.250 | 0 ==> 1 |
|  | 70 | 1 | 0.333 | 0 --> 1 |
|  | 91 | 1 | 0.333 | 0 ==> 1 |
|  | 98 | 1 | 0.500 | 1 ==> 2 |
|  | 100 | 1 | 0.500 | 1 ==> 0 |
| *Neohylomys hainanensis* | 63 | 1 | 0.500 | 0 ==> 1 |
| *Hylomys suillus* | 71 | 1 | 0.333 | 1 ==> 0 |
|  | 86 | 1 | 0.500 | 1 --> 0 |
|  | 88 | 1 | 0.500 | 0 ==> 1 |
|  | 109 | 1 | 0.500 | 0 ==> 1 |
| *Hylomys parvus* | 21 | 1 | 0.400 | 1 ==> 0 |
|  | 25 | 1 | 0.250 | 0 ==> 1 |
|  | 71 | 1 | 0.333 | 1 ==> 0 |
| Stem 4 | 1 | 1 | 0.500 | 0 ==> 1 |
|  | 2 | 1 | 1.000 | 0 ==> 1 |
|  | 4 | 2 | 0.667 | 0 ==> 2 |
|  | 6 | 1 | 0.500 | 0 ==> 1 |
|  | 7 | 1 | 0.500 | 0 ==> 1 |
|  | 10 | 1 | 0.500 | 1 ==> 2 |
|  | 17 | 1 | 1.000 | 0 ==> 1 |
|  | 18 | 1 | 1.000 | 0 ==> 1 |
|  | 21 | 1 | 0.400 | 1 ==> 2 |
|  | 22 | 1 | 0.500 | 0 ==> 1 |
|  | 24 | 1 | 1.000 | 0 ==> 1 |
|  | 27 | 1 | 1.000 | 0 ==> 1 |
|  | 28 | 1 | 1.000 | 0 ==> 1 |
|  | 30 | 1 | 1.000 | 0 ==> 1 |
|  | 33 | 1 | 1.000 | 0 ==> 1 |
|  | 36 | 1 | 1.000 | 0 --> 2 |
|  | 40 | 1 | 1.000 | 0 ==> 1 |
|  | 46 | 1 | 1.000 | 0 ==> 1 |
|  | 47 | 1 | 1.000 | 0 ==> 1 |
|  | 58 | 1 | 1.000 | 0 ==> 1 |
|  | 60 | 1 | 1.000 | 0 ==> 1 |
|  | 63 | 1 | 0.500 | 0 --> 2 |
|  | 64 | 1 | 0.500 | 1 ==> 0 |
|  | 66 | 1 | 0.500 | 0 ==> 1 |
|  | 74 | 2 | 0.667 | 0 ==> 2 |
|  | 83 | 1 | 0.500 | 0 ==> 1 |
|  | 92 | 1 | 0.571 | 2 --> 3 |
|  | 95 | 1 | 1.000 | 0 ==> 1 |
|  | 114 | 1 | 1.000 | 0 ==> 1 |
|  | 128 | 1 | 0.667 | 0 ==> 1 |
|  | 129 | 1 | 1.000 | 0 ==> 1 |
|  | 131 | 1 | 1.000 | 0 --> 1 |
|  | 132 | 1 | 1.000 | 0 ==> 1 |
|  | 133 | 1 | 1.000 | 0 ==> 3 |
|  | 134 | 1 | 0.500 | 0 --> 1 |
| Stem 5 | 3 | 1 | 1.000 | 0 ==> 1 |
|  | 30 | 1 | 1.000 | 1 ==> 2 |
|  | 34 | 1 | 1.000 | 0 ==> 1 |
|  | 127 | 1 | 1.000 | 0 ==> 1 |
|  | 130 | 1 | 1.000 | 0 --> 2 |
|  | 131 | 1 | 1.000 | 1 --> 3 |
|  | 134 | 1 | 0.500 | 1 --> 0 |
| *Paraechinus aethiopicus* | 12 | 2 | 1.000 | 1 ==> 3 |
|  | 30 | 2 | 1.000 | 2 ==> 4 |
|  | 43 | 1 | 1.000 | 0 ==> 1 |
|  | 44 | 1 | 1.000 | 0 ==> 1 |
|  | 92 | 3 | 0.571 | 3 ==> 0 |
|  | 100 | 1 | 0.500 | 1 ==> 0 |
|  | 109 | 1 | 0.500 | 0 ==> 1 |
|  | 133 | 1 | 1.000 | 3 ==> 2 |
| *Hemiechinus auritus* | 82 | 1 | 0.250 | 1 ==> 0 |
|  | 91 | 1 | 0.333 | 0 ==> 1 |
|  | 93 | 1 | 0.333 | 1 ==> 0 |
| Stem 6 | 11 | 1 | 1.000 | 1 ==> 0 |
|  | 13 | 1 | 0.500 | 1 ==> 0 |
|  | 31 | 1 | 1.000 | 0 ==> 1 |
|  | 41 | 1 | 0.500 | 0 ==> 1 |
|  | 92 | 1 | 0.571 | 3 --> 4 |
|  | 93 | 1 | 0.333 | 1 --> 0 |
|  | 97 | 1 | 1.000 | 0 --> 2 |
|  | 98 | 1 | 0.500 | 1 --> 2 |
|  | 100 | 1 | 0.500 | 1 --> 0 |
|  | 130 | 1 | 1.000 | 0 --> 1 |
|  | 131 | 1 | 1.000 | 1 --> 2 |
| *Mesechinus dauuricus* | 23 | 1 | 0.500 | 0 ==> 1 |
|  | 82 | 1 | 0.250 | 1 ==> 0 |
| *Mesechinus hughi* | 32 | 1 | 0.500 | 0 ==> 1 |
| Stem 7 | 23 | 1 | 0.500 | 0 ==> 1 |
|  | 32 | 1 | 0.500 | 0 ==> 1 |
|  | 133 | 1 | 1.000 | 3 ==> 1 |
| *Atelerix albiventris* | 21 | 1 | 0.400 | 2 ==> 1 |
|  | 25 | 1 | 0.250 | 0 ==> 1 |
|  | 29 | 1 | 1.000 | 0 ==> 1 |
|  | 73 | 1 | 1.000 | 1 ==> 0 |
|  | 113 | 1 | 0.500 | 0 ==> 1 |
|  | 128 | 1 | 0.667 | 1 ==> 2 |
| Stem 8 | 82 | 1 | 0.250 | 1 ==> 0 |
|  | 91 | 1 | 0.333 | 0 ==> 1 |
|  | 128 | 1 | 0.667 | 1 ==> 0 |
| *Erinaceus amurensis* | 93 | 1 | 0.333 | 1 ==> 0 |
|  | 110 | 1 | 0.500 | 1 ==> 0 |
|  | 112 | 1 | 0.500 | 0 ==> 1 |
| *Erinaceus europaeus* | 21 | 1 | 0.400 | 2 ==> 1 |
|  | 88 | 1 | 0.500 | 0 ==> 1 |
|  |  |  |  |  |

**7. Strict consensus morphological tree of 15 taxon based on non-dental characters.**

Tree length = 111,Consistency index (CI) = 0.7477, Homoplasy index (HI) = 0.2523, CI excluding uninformative characters = 0.7200, HI excluding uninformative characters = 0.2800, Retention index (RI) = 0.8756, Rescaled consistency index (RC) = 0.6547


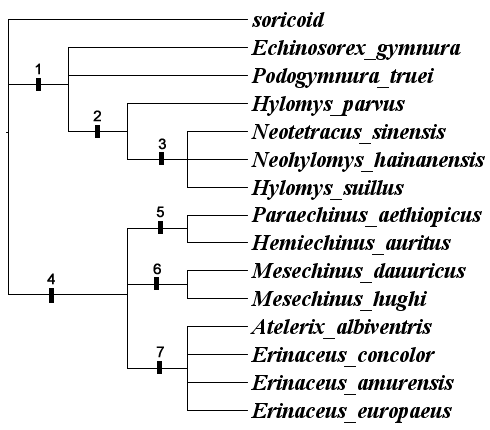


Apomorphy list

| Branch | Character | Steps | CI | Change |
| --- | --- | --- | --- | --- |
| Stem 1 | 8 | 1 | 0.500 | 0 --> 1 |
|  | 9 | 1 | 0.750 | 2 ==> 1 |
|  | 11 | 1 | 1.000 | 1 --> 2 |
|  | 41 | 1 | 0.500 | 0 ==> 1 |
|  | 42 | 1 | 0.500 | 0 --> 1 |
|  | 59 | 1 | 0.500 | 0 --> 1 |
|  | 123 | 1 | 1.000 | 0 ==> 1 |
|  | 124 | 1 | 1.000 | 0 ==> 1 |
|  | 125 | 1 | 1.000 | 0 ==> 1 |
|  | 126 | 1 | 1.000 | 0 ==> 1 |
| *Echinosorex gymnura* | 5 | 1 | 0.500 | 0 --> 1 |
|  | 7 | 1 | 0.500 | 0 ==> 1 |
|  | 8 | 1 | 0.500 | 1 --> 0 |
|  | 9 | 1 | 0.750 | 1 --> 0 |
|  | 10 | 1 | 0.500 | 1 ==> 2 |
|  | 21 | 1 | 0.400 | 1 ==> 0 |
|  | 26 | 1 | 1.000 | 0 ==> 1 |
|  | 35 | 1 | 0.500 | 1 ==> 0 |
|  | 42 | 1 | 0.500 | 1 --> 0 |
|  | 59 | 1 | 0.500 | 1 --> 0 |
|  | 61 | 1 | 0.500 | 0 --> 1 |
|  | 121 | 1 | 1.000 | 0 --> 1 |
|  | 122 | 1 | 1.000 | 0 --> 1 |
|  | 135 | 1 | 0.500 | 0 --> 1 |
| *Podogymnura truei* | 5 | 1 | 0.500 | 0 --> 1 |
|  | 9 | 1 | 0.750 | 1 --> 0 |
|  | 10 | 1 | 0.500 | 1 ==> 0 |
|  | 22 | 1 | 0.500 | 0 ==> 1 |
|  | 25 | 1 | 0.250 | 0 ==> 1 |
|  | 61 | 1 | 0.500 | 0 --> 1 |
|  | 135 | 1 | 0.500 | 0 --> 1 |
| Stem 2 | 4 | 1 | 0.667 | 0 ==> 1 |
|  | 19 | 1 | 1.000 | 0 ==> 1 |
|  | 121 | 1 | 1.000 | 0 --> 1 |
|  | 122 | 1 | 1.000 | 0 --> 1 |
| Stem 3 | 6 | 1 | 0.500 | 0 ==> 1 |
|  | 14 | 1 | 1.000 | 0 ==> 1 |
|  | 15 | 1 | 1.000 | 1 ==> 0 |
|  | 16 | 1 | 1.000 | 0 ==> 1 |
| *Neotetracus sinensis* | 1 | 1 | 0.500 | 0 ==> 1 |
|  | 25 | 1 | 0.250 | 0 ==> 1 |
| *Hylomys parvus* | 21 | 1 | 0.400 | 1 ==> 0 |
|  | 25 | 1 | 0.250 | 0 ==> 1 |
| Stem 4 | 1 | 1 | 0.500 | 0 ==> 1 |
|  | 2 | 1 | 1.000 | 0 ==> 1 |
|  | 4 | 2 | 0.667 | 0 ==> 2 |
|  | 6 | 1 | 0.500 | 0 ==> 1 |
|  | 7 | 1 | 0.500 | 0 ==> 1 |
|  | 10 | 1 | 0.500 | 1 ==> 2 |
|  | 17 | 1 | 1.000 | 0 ==> 1 |
|  | 18 | 1 | 1.000 | 0 ==> 1 |
|  | 21 | 1 | 0.400 | 1 ==> 2 |
|  | 22 | 1 | 0.500 | 0 ==> 1 |
|  | 24 | 1 | 1.000 | 0 ==> 1 |
|  | 27 | 1 | 1.000 | 0 ==> 1 |
|  | 28 | 1 | 1.000 | 0 ==> 1 |
|  | 30 | 1 | 1.000 | 0 ==> 1 |
|  | 33 | 1 | 1.000 | 0 ==> 1 |
|  | 36 | 1 | 1.000 | 0 --> 2 |
|  | 40 | 1 | 1.000 | 0 ==> 1 |
|  | 46 | 1 | 1.000 | 0 ==> 1 |
|  | 47 | 1 | 1.000 | 0 ==> 1 |
|  | 58 | 1 | 1.000 | 0 ==> 1 |
|  | 60 | 1 | 1.000 | 0 ==> 1 |
|  | 128 | 1 | 0.500 | 0 --> 1 |
|  | 129 | 1 | 1.000 | 0 ==> 1 |
|  | 131 | 1 | 1.000 | 0 --> 1 |
|  | 132 | 1 | 1.000 | 0 ==> 1 |
|  | 133 | 1 | 1.000 | 0 ==> 3 |
|  | 134 | 1 | 0.500 | 0 --> 1 |
| Stem 5 | 3 | 1 | 1.000 | 0 ==> 1 |
|  | 30 | 1 | 1.000 | 1 ==> 2 |
|  | 34 | 1 | 1.000 | 0 ==> 1 |
|  | 127 | 1 | 1.000 | 0 ==> 1 |
|  | 130 | 1 | 1.000 | 0 --> 2 |
|  | 131 | 1 | 1.000 | 1 --> 3 |
|  | 134 | 1 | 0.500 | 1 --> 0 |
| *Paraechinus aethiopicus* | 12 | 2 | 1.000 | 1 ==> 3 |
|  | 30 | 2 | 1.000 | 2 ==> 4 |
|  | 43 | 1 | 1.000 | 0 ==> 1 |
|  | 44 | 1 | 1.000 | 0 ==> 1 |
|  | 133 | 1 | 1.000 | 3 ==> 2 |
| Stem 6 | 11 | 1 | 1.000 | 1 ==> 0 |
|  | 13 | 1 | 0.500 | 1 ==> 0 |
|  | 31 | 1 | 1.000 | 0 ==> 1 |
|  | 41 | 1 | 0.500 | 0 ==> 1 |
|  | 130 | 1 | 1.000 | 0 --> 1 |
|  | 131 | 1 | 1.000 | 1 --> 2 |
| *Mesechinus dauuricus* | 23 | 1 | 0.500 | 0 ==> 1 |
| *Mesechinus hughi* | 32 | 1 | 0.500 | 0 ==> 1 |
| Stem 7 | 23 | 1 | 0.500 | 0 ==> 1 |
|  | 32 | 1 | 0.500 | 0 ==> 1 |
|  | 128 | 1 | 0.500 | 1 --> 0 |
|  | 133 | 1 | 1.000 | 3 ==> 1 |
| *Atelerix albiventris* | 21 | 1 | 0.400 | 2 ==> 1 |
|  | 25 | 1 | 0.250 | 0 ==> 1 |
|  | 29 | 1 | 1.000 | 0 ==> 1 |
|  | 128 | 2 | 0.500 | 0 ==> 2 |
| *Erinaceus europaeus* | 21 | 1 | 0.400 | 2 ==> 1 |

**8. Strict consensus morphological tree of 15 taxon based on only dental characters.**

Tree length = 51, Consistency index (CI) = 0.6667, Homoplasy index (HI) = 0.3333, CI excluding uninformative characters = 0.6136, HI excluding uninformative characters = 0.3864, Retention index (RI) = 0.7385, Rescaled consistency index (RC) = 0.4923


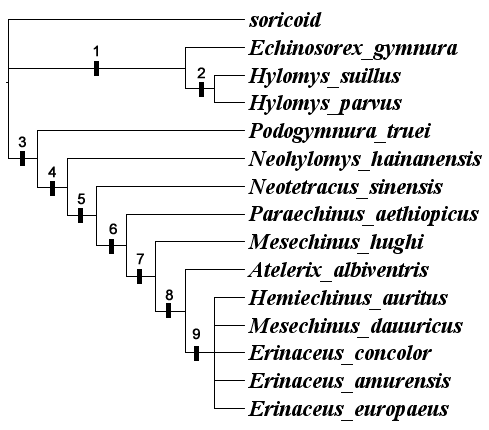


**Apomorphy list**

| Branch | Character | Steps | CI | Change |
| --- | --- | --- | --- | --- |
| *Echinosorex gymnura* | 63 | 1 | 1.000 | 1 --> 0 |
|  | 64 | 1 | 0.500 | 1 ==> 0 |
|  | 70 | 1 | 0.500 | 1 --> 0 |
|  | 82 | 1 | 0.500 | 1 ==> 0 |
|  | 86 | 1 | 1.000 | 1 --> 0 |
|  | 92 | 2 | 0.667 | 0 ==> 2 |
|  | 100 | 1 | 0.667 | 0 --> 1 |
|  | 112 | 1 | 0.500 | 0 --> 1 |
| Stem 2 | 63 | 1 | 1.000 | 1 --> 0 |
|  | 70 | 1 | 0.500 | 1 --> 0 |
|  | 83 | 1 | 0.500 | 0 ==> 1 |
|  | 86 | 1 | 1.000 | 1 --> 0 |
|  | 88 | 1 | 0.500 | 0 --> 1 |
|  | 100 | 1 | 0.667 | 0 --> 1 |
|  | 109 | 1 | 0.500 | 0 --> 1 |
| Stem 3 | 71 | 1 | 1.000 | 0 ==> 1 |
| *Podogymnura truei* | 66 | 1 | 0.500 | 0 ==> 1 |
| Stem 4 | 74 | 1 | 1.000 | 0 ==> 1 |
|  | 83 | 1 | 0.500 | 0 ==> 1 |
| Stem 5 | 63 | 1 | 1.000 | 1 ==> 2 |
| *Neotetracus sinensis* | 91 | 1 | 0.500 | 0 ==> 1 |
|  | 98 | 1 | 0.500 | 1 ==> 2 |
| Stem 6 | 64 | 1 | 0.500 | 1 ==> 0 |
|  | 66 | 1 | 0.500 | 0 ==> 1 |
|  | 74 | 1 | 1.000 | 1 ==> 2 |
|  | 95 | 1 | 1.000 | 0 ==> 1 |
|  | 114 | 1 | 1.000 | 0 ==> 1 |
| *Paraechinus aethiopicus* | 109 | 1 | 0.500 | 0 ==> 1 |
| Stem 7 | 92 | 3 | 0.667 | 0 ==> 3 |
|  | 93 | 1 | 0.333 | 1 --> 0 |
| Stem 8 | 100 | 1 | 0.667 | 0 ==> 1 |
| Stem 9 | 82 | 1 | 0.500 | 1 ==> 0 |
|  | 91 | 1 | 0.500 | 0 ==> 1 |
| *Erinaceus amurensis* | 110 | 1 | 0.500 | 1 ==> 0 |
|  | 112 | 1 | 0.500 | 0 ==> 1 |
| *Erinaceus europaeus* | 88 | 1 | 0.500 | 0 ==> 1 |
|  | 93 | 1 | 0.333 | 0 --> 1 |
| *Atelerix albiventris* | 73 | 1 | 1.000 | 1 ==> 0 |
|  | 93 | 1 | 0.333 | 0 --> 1 |
|  | 113 | 1 | 0.500 | 0 ==> 1 |
| *Mesechinus hughi* | 92 | 1 | 0.667 | 3 ==> 4 |
|  | 97 | 1 | 1.000 | 0 ==> 2 |
|  | 98 | 1 | 0.500 | 1 ==> 2 |
| *Neohylomys hainanensis* | 70 | 1 | 0.500 | 1 ==> 0 |

**9. Combined data tree 1 including 14 erinaceids species**

Tree length (only 112 morphological characters were considered) = 175, Consistency index (CI) = 0.6514, Homoplasy index (HI) = 0.3486, CI excluding uninformative characters = 0.6474, HI excluding uninformative characters = 0.3526, Retention index (RI) = 0.9270, Rescaled consistency index (RC) = 0.6039


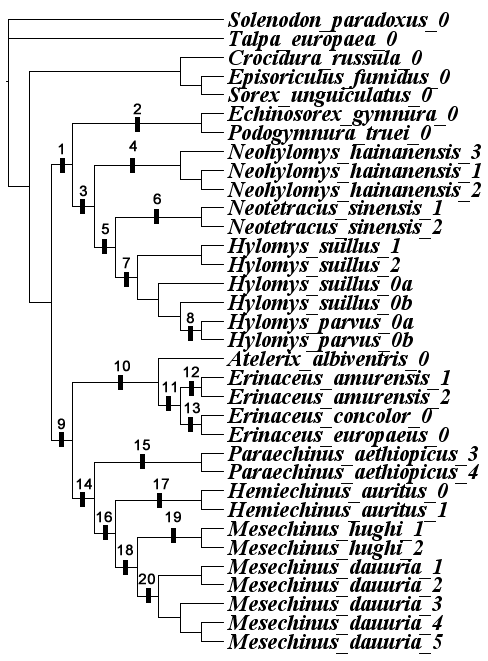


**Apomorphy list**

| Branch | Character | Steps | CI | Change |
| --- | --- | --- | --- | --- |
| Stem 9 | 9 | 1 | 1.000 | 3 --> 2 |
|  | 10 | 1 | 0.500 | 0 ==> 1 |
|  | 12 | 1 | 1.000 | 0 --> 1 |
|  | 13 | 1 | 0.500 | 0 --> 1 |
|  | 20 | 1 | 0.500 | 0 --> 1 |
|  | 35 | 1 | 0.500 | 0 --> 1 |
|  | 45 | 1 | 1.000 | 0 --> 1 |
|  | 51 | 1 | 1.000 | 0 --> 1 |
|  | 54 | 1 | 1.000 | 0 --> 1 |
|  | 71 | 1 | 0.500 | 0 --> 1 |
|  | 89 | 1 | 1.000 | 0 --> 1 |
|  | 90 | 1 | 1.000 | 0 --> 1 |
|  | 94 | 1 | 1.000 | 0 --> 2 |
|  | 96 | 1 | 1.000 | 1 --> 0 |
|  | 110 | 1 | 0.500 | 0 --> 1 |
|  | 113 | 1 | 0.500 | 1 --> 0 |
|  | 130 | 1 | 1.000 | 0 --> 2 |
|  | 133 | 1 | 1.000 | 0 --> 1 |
| Stem 10 | 1 | 1 | 0.500 | 0 --> 1 |
|  | 2 | 1 | 1.000 | 0 --> 1 |
|  | 4 | 2 | 0.667 | 0 --> 2 |
|  | 6 | 1 | 0.333 | 0 --> 1 |
|  | 7 | 1 | 0.500 | 0 --> 1 |
|  | 10 | 1 | 0.500 | 1 --> 2 |
|  | 11 | 1 | 1.000 | 3 --> 1 |
|  | 17 | 1 | 1.000 | 0 --> 1 |
|  | 18 | 1 | 1.000 | 0 --> 1 |
|  | 21 | 1 | 0.500 | 1 --> 2 |
|  | 22 | 1 | 0.500 | 0 --> 1 |
|  | 24 | 1 | 1.000 | 0 --> 1 |
|  | 27 | 1 | 1.000 | 0 --> 1 |
|  | 28 | 1 | 1.000 | 0 --> 1 |
|  | 30 | 1 | 0.800 | 0 --> 1 |
|  | 33 | 1 | 1.000 | 0 --> 1 |
|  | 36 | 1 | 1.000 | 0 --> 2 |
|  | 40 | 1 | 1.000 | 0 --> 1 |
|  | 46 | 1 | 1.000 | 0 --> 1 |
|  | 47 | 1 | 1.000 | 0 --> 1 |
|  | 58 | 1 | 1.000 | 0 --> 1 |
|  | 60 | 1 | 1.000 | 0 --> 1 |
|  | 63 | 1 | 0.500 | 1 --> 2 |
|  | 64 | 1 | 0.500 | 1 --> 0 |
|  | 66 | 1 | 0.500 | 0 --> 1 |
|  | 74 | 2 | 0.500 | 0 --> 2 |
|  | 83 | 1 | 0.500 | 0 --> 1 |
|  | 95 | 1 | 1.000 | 0 --> 1 |
|  | 100 | 1 | 0.500 | 2 --> 1 |
|  | 114 | 1 | 1.000 | 0 --> 1 |
|  | 129 | 1 | 1.000 | 0 --> 1 |
|  | 132 | 1 | 1.000 | 0 --> 1 |
| *Atelerix albiventris_0* | 21 | 1 | 0.500 | 2 --> 1 |
|  | 23 | 1 | 0.500 | 0 --> 1 |
|  | 25 | 1 | 0.250 | 0 ==> 1 |
|  | 29 | 1 | 1.000 | 0 ==> 1 |
|  | 32 | 1 | 0.500 | 0 --> 1 |
|  | 113 | 1 | 0.500 | 0 ==> 1 |
|  | 128 | 2 | 0.667 | 0 ==> 2 |
|  | 130 | 1 | 1.000 | 2 --> 0 |
|  | 133 | 1 | 1.000 | 1 --> 0 |
|  | 134 | 1 | 0.500 | 0 --> 1 |
| Stem 11 | 23 | 1 | 0.500 | 0 --> 1 |
|  | 32 | 1 | 0.500 | 0 --> 1 |
|  | 82 | 1 | 0.250 | 1 ==> 0 |
|  | 91 | 1 | 0.333 | 0 ==> 1 |
|  | 92 | 1 | 0.571 | 2 --> 3 |
|  | 130 | 1 | 1.000 | 2 --> 0 |
|  | 133 | 1 | 1.000 | 1 --> 0 |
|  | 134 | 1 | 0.500 | 0 --> 1 |
| Stem 12 | 93 | 1 | 0.500 | 1 ==> 0 |
|  | 110 | 1 | 0.500 | 1 ==> 0 |
|  | 112 | 1 | 0.500 | 0 ==> 1 |
|  | 131 | 1 | 1.000 | 0 ==> 1 |
| Stem 13 | 88 | 1 | 0.500 | 0 --> 1 |
| *Erinaceus europaeus_0* | 20 | 1 | 0.500 | 1 ==> 0 |
| Stem 14 | 1 | 1 | 0.500 | 0 --> 1 |
|  | 2 | 1 | 1.000 | 0 --> 1 |
|  | 3 | 1 | 0.500 | 0 --> 1 |
|  | 4 | 2 | 0.667 | 0 --> 2 |
|  | 6 | 1 | 0.333 | 0 --> 1 |
|  | 7 | 1 | 0.500 | 0 --> 1 |
|  | 10 | 1 | 0.500 | 1 --> 2 |
|  | 11 | 1 | 1.000 | 3 --> 1 |
|  | 17 | 1 | 1.000 | 0 --> 1 |
|  | 18 | 1 | 1.000 | 0 --> 1 |
|  | 21 | 1 | 0.500 | 1 --> 2 |
|  | 22 | 1 | 0.500 | 0 --> 1 |
|  | 24 | 1 | 1.000 | 0 --> 1 |
|  | 27 | 1 | 1.000 | 0 --> 1 |
|  | 28 | 1 | 1.000 | 0 --> 1 |
|  | 30 | 2 | 0.800 | 0 --> 2 |
|  | 33 | 1 | 1.000 | 0 --> 1 |
|  | 34 | 1 | 0.500 | 0 --> 1 |
|  | 36 | 1 | 1.000 | 0 --> 2 |
|  | 40 | 1 | 1.000 | 0 --> 1 |
|  | 46 | 1 | 1.000 | 0 --> 1 |
|  | 47 | 1 | 1.000 | 0 --> 1 |
|  | 58 | 1 | 1.000 | 0 --> 1 |
|  | 60 | 1 | 1.000 | 0 --> 1 |
|  | 63 | 1 | 0.500 | 1 --> 2 |
|  | 64 | 1 | 0.500 | 1 --> 0 |
|  | 66 | 1 | 0.500 | 0 --> 1 |
|  | 74 | 2 | 0.500 | 0 --> 2 |
|  | 83 | 1 | 0.500 | 0 --> 1 |
|  | 95 | 1 | 1.000 | 0 --> 1 |
|  | 100 | 1 | 0.500 | 2 --> 0 |
|  | 114 | 1 | 1.000 | 0 --> 1 |
|  | 127 | 1 | 0.500 | 0 --> 1 |
|  | 128 | 1 | 0.667 | 0 --> 1 |
|  | 129 | 1 | 1.000 | 0 --> 1 |
|  | 132 | 1 | 1.000 | 0 --> 1 |
| Stem 16 | 82 | 1 | 0.250 | 1 --> 0 |
|  | 92 | 1 | 0.571 | 2 --> 3 |
|  | 93 | 1 | 0.500 | 1 ==> 0 |
|  | 133 | 1 | 1.000 | 1 --> 2 |
| Stem 17 | 91 | 1 | 0.333 | 0 ==> 1 |
|  | 100 | 1 | 0.500 | 0 --> 1 |
| Stem 18 | 3 | 1 | 0.500 | 1 --> 0 |
|  | 13 | 1 | 0.500 | 1 ==> 0 |
|  | 30 | 1 | 0.800 | 2 --> 1 |
|  | 31 | 1 | 1.000 | 0 ==> 1 |
|  | 34 | 1 | 0.500 | 1 --> 0 |
|  | 41 | 1 | 0.500 | 0 ==> 1 |
|  | 62 | 1 | 0.500 | 1 --> 0 |
|  | 92 | 1 | 0.571 | 3 --> 4 |
|  | 97 | 1 | 1.000 | 0 --> 2 |
|  | 98 | 1 | 0.500 | 1 --> 2 |
|  | 127 | 1 | 0.500 | 1 --> 0 |
|  | 130 | 1 | 1.000 | 2 --> 1 |
|  | 134 | 1 | 0.500 | 0 --> 1 |
| Stem 20 | 23 | 1 | 0.500 | 0 ==> 1 |
| Stem 19 | 11 | 1 | 1.000 | 1 ==> 0 |
|  | 32 | 1 | 0.500 | 0 ==> 1 |
|  | 73 | 1 | 0.500 | 0 ==> 1 |
|  | 82 | 1 | 0.250 | 0 --> 1 |
| Stem 15 | 12 | 2 | 1.000 | 1 ==> 3 |
|  | 30 | 2 | 0.800 | 2 ==> 4 |
|  | 43 | 1 | 1.000 | 0 ==> 1 |
|  | 44 | 1 | 1.000 | 0 ==> 1 |
|  | 92 | 2 | 0.571 | 2 ==> 0 |
|  | 109 | 1 | 0.500 | 0 ==> 1 |
| Stem 1 | 8 | 1 | 0.500 | 0 --> 1 |
|  | 9 | 2 | 1.000 | 3 ==> 1 |
|  | 10 | 1 | 0.500 | 0 --> 1 |
|  | 11 | 1 | 1.000 | 3 --> 2 |
|  | 12 | 1 | 1.000 | 0 --> 1 |
|  | 13 | 1 | 0.500 | 0 --> 1 |
|  | 20 | 1 | 0.500 | 0 --> 1 |
|  | 35 | 1 | 0.500 | 0 --> 1 |
|  | 41 | 1 | 0.500 | 0 ==> 1 |
|  | 42 | 1 | 0.500 | 0 --> 1 |
|  | 45 | 1 | 1.000 | 0 --> 1 |
|  | 51 | 1 | 1.000 | 0 --> 1 |
|  | 54 | 1 | 1.000 | 0 --> 1 |
|  | 59 | 1 | 0.500 | 0 --> 1 |
|  | 70 | 1 | 0.333 | 1 --> 0 |
|  | 71 | 1 | 0.500 | 0 --> 1 |
|  | 86 | 1 | 0.500 | 1 --> 0 |
|  | 89 | 1 | 1.000 | 0 --> 1 |
|  | 90 | 1 | 1.000 | 0 --> 1 |
|  | 94 | 1 | 1.000 | 0 --> 2 |
|  | 96 | 1 | 1.000 | 1 --> 0 |
|  | 100 | 1 | 0.500 | 2 --> 1 |
|  | 110 | 1 | 0.500 | 0 --> 1 |
|  | 113 | 1 | 0.500 | 1 --> 0 |
|  | 121 | 1 | 1.000 | 0 ==> 1 |
|  | 122 | 1 | 1.000 | 0 ==> 1 |
|  | 123 | 1 | 1.000 | 0 ==> 1 |
|  | 124 | 1 | 1.000 | 0 ==> 1 |
|  | 125 | 1 | 1.000 | 0 ==> 1 |
|  | 126 | 1 | 1.000 | 0 ==> 1 |
| Stem 2 | 5 | 1 | 1.000 | 0 ==> 1 |
|  | 9 | 1 | 1.000 | 1 ==> 0 |
|  | 61 | 1 | 1.000 | 0 ==> 1 |
|  | 112 | 1 | 0.500 | 0 --> 1 |
|  | 135 | 1 | 1.000 | 0 ==> 1 |
| *Echinosorex gymnura_0* | 7 | 1 | 0.500 | 0 ==> 1 |
|  | 8 | 1 | 0.500 | 1 --> 0 |
|  | 10 | 1 | 0.500 | 1 ==> 2 |
|  | 21 | 1 | 0.500 | 1 ==> 0 |
|  | 26 | 1 | 1.000 | 0 ==> 1 |
|  | 35 | 1 | 0.500 | 1 ==> 0 |
|  | 42 | 1 | 0.500 | 1 --> 0 |
|  | 59 | 1 | 0.500 | 1 --> 0 |
|  | 63 | 1 | 0.500 | 1 ==> 0 |
|  | 64 | 1 | 0.500 | 1 ==> 0 |
|  | 82 | 1 | 0.250 | 1 ==> 0 |
| *Podogymnura truei_0* | 10 | 1 | 0.500 | 1 ==> 0 |
|  | 22 | 1 | 0.500 | 0 ==> 1 |
|  | 25 | 1 | 0.250 | 0 ==> 1 |
|  | 66 | 1 | 0.500 | 0 ==> 1 |
|  | 70 | 1 | 0.333 | 0 --> 1 |
| Stem 3 | 4 | 1 | 0.667 | 0 --> 1 |
|  | 6 | 1 | 0.333 | 0 --> 1 |
|  | 14 | 1 | 0.500 | 0 ==> 1 |
|  | 15 | 1 | 0.500 | 1 ==> 0 |
|  | 16 | 1 | 0.500 | 0 ==> 1 |
|  | 19 | 1 | 1.000 | 0 ==> 1 |
|  | 74 | 1 | 0.500 | 0 --> 1 |
|  | 83 | 1 | 0.500 | 0 --> 1 |
|  | 92 | 2 | 0.571 | 2 --> 0 |
| Stem 7 | 71 | 1 | 0.500 | 1 ==> 0 |
|  | 74 | 1 | 0.500 | 1 --> 0 |
|  | 88 | 1 | 0.500 | 0 ==> 1 |
|  | 109 | 1 | 0.500 | 0 ==> 1 |
| Stem 8 | 6 | 1 | 0.333 | 1 ==> 0 |
|  | 14 | 1 | 0.500 | 1 ==> 0 |
|  | 15 | 1 | 0.500 | 0 ==> 1 |
|  | 16 | 1 | 0.500 | 1 ==> 0 |
|  | 21 | 1 | 0.500 | 1 ==> 0 |
|  | 25 | 1 | 0.250 | 0 ==> 1 |
|  | 63 | 1 | 0.500 | 1 ==> 0 |
|  | 73 | 1 | 0.500 | 0 ==> 1 |
| Stem 6 | 1 | 1 | 0.500 | 0 ==> 1 |
|  | 25 | 1 | 0.250 | 0 ==> 1 |
|  | 62 | 1 | 0.500 | 1 ==> 0 |
|  | 63 | 1 | 0.500 | 1 ==> 0 |
|  | 70 | 1 | 0.333 | 0 --> 1 |
|  | 86 | 1 | 0.500 | 0 --> 1 |
|  | 91 | 1 | 0.333 | 0 ==> 1 |
|  | 98 | 1 | 0.500 | 1 ==> 2 |
|  | 100 | 1 | 0.500 | 1 ==> 0 |
